# Global sensitivity analysis to enhance the transparency and rigour of energy system optimisation modelling

**DOI:** 10.12688/openreseurope.15461.1

**Published:** 2023-02-13

**Authors:** William Usher, Trevor Barnes, Nandi Moksnes, Taco Niet

**Affiliations:** 1 Energy Technology, KTH Royal Institute of Technology, Stockholm, Sweden; 2 School of Sustainable Energy Engineering, Simon Fraser University, Vancouver, Canada

**Keywords:** Global Sensitivity Analysis, Energy System Optimisation Modelling, Uncertainty, Transparency

## Abstract

**Background: **Energy system optimisation models (ESOMs) are commonly used to support long-term planning at national, regional, or continental scales. The importance of recognising uncertainty in energy system modelling is regularly commented on but there is little practical guidance on how to best incorporate existing techniques, such as global sensitivity analysis, despite some good applications in the literature.

**Methods: **In this paper, we provide comprehensive guidelines for conducting a global sensitivity analysis of an ESOM, aiming to remove barriers to adopting this approach. With a pedagogical intent, we begin by exploring why you should conduct a global sensitivity analysis. We then describe how to implement a global sensitivity analysis using the Morris method in an ESOM using a sequence of simple illustrative models built using the Open Source energy Modelling System (OSeMOSYS) framework, followed by a realistic example.

**Results: **Results show that the global sensitivity analysis identifies influential parameters that drive results in the simple and realistic models, and identifies uninfluential parameters which can be ignored or fixed. We show that global sensitivity analysis can be applied to ESOMs with relative ease using freely available open-source tools. The results replicate the findings of best-practice studies from the field demonstrating the importance of including all parameters in the analysis and avoiding a narrow focus on particular parameters such as technology costs.

**Conclusions:** The results highlight the benefits of performing a global sensitivity analysis for the design of energy system optimisation scenarios. We discuss how the results can be interpreted and used to enhance the transparency and rigour of energy system modelling studies.

## Plain language summary

Energy system models are used for planning national energy systems. There is little practical guidance on how to best incorporate existing techniques that help plan energy systems under uncertainty. Given the large costs of energy infrastructure and the important role it plays in the economy, it is essential to improve the way in which we plan our energy systems.

One technique to manage uncertainty in models is called global sensitivity analysis. In this paper, we provide guidelines to conduct a global sensitivity analysis of an energy system model. We use simple energy system models to show how global sensitivity analysis works. We then use a more complex and realistic model to show what sort of results could be generated in reality.

The paper shows that using global sensitivity analysis provides added insights to those who develop and use energy system models. The approach can identify which uncertain parameters most influence the model results. This allows researchers to focus their effort on better understanding these parameters, increasing the robustness of their work, and increasing confidence in the models and their results.

## Introduction

Energy system optimisation models (ESOMs) are used to develop insights into the plausible future evolution of the energy system for planning and policy support purposes. The use of ESOMs sits within the context of post-normal science ( Ravetz, 1999) - the energy transition required to meet climate goals implies a fundamental restructuring of social, economic and infrastructure systems (high-stakes), while planning the transition takes place under severe system-wide uncertainties. To manage this uncertainty, ESOMs are usually used in combination with scenario analysis, where a narrative or storyline is quantified using the ESOM parameters to explore plausible futures ( DeCarolis *et al.*, 2017). One drawback with the implementation of scenario analysis is that studies tend to explore only a small number of dimensions in the model input space, increasing the risk of systematic bias in the results reported in the literature ( Morgan & Keith, 2008). And energy modellers should better explain the key drivers of model results, to avoid misinterpretation of their results ( Braunreiter & Blumer, 2018). While various approaches are currently used to mitigate the risk of systematic bias, including model comparison studies, and calls for exploring extreme scenarios, the adoption of global sensitivity analysis (GSA) is under-represented.

In this paper, we demonstrate the utility of GSA for the ESOM community. A recent position paper states: “ *We believe that [sensitivity analysis] science must formally become an integral part of systems analysis in general, and mathematical modelling in particular, to unleash the capabilities of [sensitivity analysis] for addressing emerging problems in engineering, technology, the natural and social sciences, and human-natural systems*” ( Razavi *et al.*, 2021, 2). To date, this is not the case in the ESOM community. To address this, we aim to clearly demonstrate, in the context of ESOMs, the benefits of GSA, the challenges associated with it, and how to interpret the results.

To motivate this paper, we first outline the key characteristics of ESOMs, and explain how these characteristics influence the results obtained from ESOMs and why methods are needed to obtain a deeper understanding of model behaviour. We then introduce GSA and summarise the benefits and challenges of applying GSA to ESOMs. Following this introduction, we summarise the relatively small literature in which GSA has been applied to ESOMs. We describe our methodology to conduct the GSA, including the design of a sequence of ESOMs of increasing complexity, beginning with a very simple example and finishing with an example using the Global Open Source Energy Modelling System (OSeMOSYS Global) ( Barnes *et al.*, 2022). We present the GSA results and provide explanations of how to interpret the results in the context of ESOMs. The tone of the paper is intentionally pedagogical, aligned with our intention to provide a reference point for ESOM practitioners, and a gateway into the practical application of sensitivity analysis.

### Energy system optimisation models

ESOMs can be thought of as an *outcome in, policy pathway out* model, in comparison to a simulation model which can be characterised as a *policy pathway in, outcome out* approach. The desired policy outcome, such as a cumulative or annual emission cap, can be easily implemented by an ESOM analyst through the imposition of constraints upon the system of equations. Through the minimisation of a cost-based objective function, the ESOM calculates the least-cost energy system subject to these constraints ( Howells *et al.*, 2011). ESOMs are typically implemented in an optimisation framework as a linear programme (LP) or mixed-integer programme (MIP). LPs are guaranteed to converge to a global optimum but make a few simplifying assumptions. MIPs are solved approximately and are more computationally demanding, but allow more realistic phenomena to be captured, such as minimum power-station capacities ( DeCarolis *et al.*, 2017).

ESOMs are prognostic models, with several core structural attributes ( Table 1) that, like all models, do not fully reflect reality but provide a set of convenient behaviours. The ability of ESOMs to generate optimal least-cost pathways for decarbonising a national energy system is useful for researchers and policymakers who wish to gain insights into the myriad possibilities, endless combinations of technologies, and the complex trade-offs across sectors, technologies, fuels and resources ( Trutnevyte, 2016). The assumption of perfect foresight provides an opportunity to explore trade-offs between decisions made now and those in the future ( Fuso Nerini *et al.*, 2017). The assumption of a single all-seeing decision maker shows the opportunities for collaboration across or trade-offs between sectors and trade between countries and regions ( DeCarolis *et al.*, 2017, 188).

**Table 1.** Core assumptions in energy system optimisation models.

Assumption	Description
Rational decision making	All decisions are made to minimize the objective function, often total-discounted cost. This provides a “best-case” or aspirational sequence of cost-optimal decisions
Perfect foresight	An artefact of the linear-programming method. Decisions in each timestep of the model are made with perfect knowledge of future outcomes and the consequences of earlier decisions
Single decision maker	No actor-specific preferences or behaviours are represented, and all “decisions” made in the model are in the service of minimising the objective function

Relaxations of each of these assumptions have been explored in various studies. Modelling to generate alternatives explores near-optimal solutions ( DeCarolis, 2011). Myopic and stochastic programming variants of ESOMs explore near-term decision-making when the future is uncertain ( William Usher & Strachan, 2012). Various methods have been used to represent preferences, including using technology- and sector-specific discount- or hurdle- rates to represent the non-tangible costs perceived by actors ( García-Gusano *et al.*, 2016).

When ESOMs model parameters change, for example, a technology cost is increased, or a new low-carbon energy technology is added, it is often not obvious how this will change the results. For example, there are results from ESOMs showing the highly non-linear response of system costs to a linear decrease in carbon emissions ( Jayadev *et al.*, 2020, 14), or system costs to changes in transmission line expansion ( Schlachtberger *et al.*, 2017). This perhaps counter-intuitive behaviour is due to the complex interaction of environmental, financial, technology, resource and operational constraints. This complexity is compounded by ESOMs data-intensity, and the complexity of the potential chains of technologies and commodities represented in the energy system. We briefly explore these two aspects below.

ESOMs are very data-intensive, with many individual assumptions, and consequently a very large input space. With the recognition of the importance of higher spatial and temporal resolutions in the design of renewable energy systems, ESOMs are getting larger, compounding the data problem, and increasing the number of sources of uncertainty and potential for error ( Saltelli, 2019). In response, researchers explore techniques to reduce the dimensionality of ESOMs. For example, clustering algorithms are applied to identify a subset of representative time slices that minimise the error of modelling at a coarser temporal resolution, essential when looking across energy demand and supply of variable renewable energy sources, such as wind and solar ( Marcy *et al.*, 2022). In this study we show how GSA can inform dimensionality reduction of ESOMs by indicating which parameters do not influence the results of the model.

The parameterisation of data-intensive ESOMs, such as OSeMOSYS, are related to the portfolio of available supply (energy conversion) and demand (energy service) technologies, fuels and resources ( Howells *et al.*, 2011). Technologies are parameterised with input and output fuels, capital, variable and fixed costs, efficiencies, capacity factors, and economic and technical lifetimes. Fossil resources are represented by costs, extraction limits and emission factors . The availability of technologies and resources may be constrained by growth- or build- rates and start-year parameters. Due to the behaviour of optimisation models, some of this technology data can be redundant because ESOMs choose the cheapest supply technologies to meet demand (subject to constraints on resources, build rates *etc.*), so parameters relating to more expensive technologies may not appear to influence the results when assumptions are held at central values and do not explore extremes ( McCollum *et al.*, 2020).

There can be strong interactions between assumptions, governed by the path-dependent structure of the system; for example, between fuel prices, technology costs, and constraints on resources, particularly when models are operating near their limits, such as under very deep decarbonisation trajectories or high demands which can be explored using modelling to generate alternatives ( DeCarolis, 2011; Pickering *et al.*, 2022). This can also result in strong non-linear behaviour ^1^, where costly technologies suddenly appear in the results due to factors such as increased demands for electricity or a cost reduction in novel fuels, combined with constraints on competing technologies and resources. This is one of the justifications for exploring extreme scenarios ( McCollum *et al.*, 2020; Trutnevyte *et al.*, 2016), as it helps reveal models that are overly constrained and cannot respond to such scenarios in a plausibly realistic fashion.

As the gap increases between reality and ambitious climate goals, it becomes more important for the users and developers of ESOMs to interrogate the behaviour of their models. GSA provides a complementary approach to existing methods, such as model comparison and exploration of extreme scenarios, to support this endeavour.

### Introduction to global sensitivity analysis

GSA is “ *the study of how uncertainty in the output of a model…can be apportioned to different sources of uncertainty in the model input*” ( Saltelli *et al.*, 2004, 45). An introduction to GSA can be found in Saltelli’s works, including the book “Global Sensitivity Analysis: The Primer” ( Saltelli *et al.*, 2008).

According to ( Saltelli *et al.*, 2008) in a typical analysis, a researcher first quantifies the distribution and ranges of each uncertain input parameter, propagates the uncertainty through the model, using a Monte Carlo sampling approach or equivalent, and finally conducts a GSA. This prior step is often called uncertainty analysis and is a precursor and separate process to GSA. We do not cover uncertainty quantification in this paper and instead focus on sensitivity analysis (SA).

In a local SA, only one input dimension is explored while holding all other dimensions at a fixed value. This is typically how scenario analyses using ESOMs are conducted ( DeCarolis *et al.*, 2017). An ESOM scenario represents a single point in the multi-dimensional input space to the model. Scenarios are often complemented with “sensitivity scenarios”, where one or more parameter values are varied around the input space of the first scenario. These sensitivity scenarios explore only a small and narrow cluster of dimensions within the input space ( Saltelli *et al.*, 2019).

For a simple model (linear and additive), a local SA, or the simple computation of derivatives, would suffice to inform which input most influences an output. The behaviour of an additive model can be trivially decomposed to the individual effects of the inputs. And if the behaviour of each input is linear, then a derivative computed anywhere in the parameter’s range captures the influence of the parameter upon the output. But for most models, where input parameters interact (influence one another) and have non-linear effects on the results (their effect on the output changes in magnitude over the parameter range), as we have established is the case for ESOMs, then a more comprehensive approach is required ( Saltelli & Annoni, 2010).

The results from a GSA cover the entire input space of a model. The global approach ensures that measures of sensitivity account for both direct effects of a parameter – how important that parameter is alone in influencing the results – as well as interaction effects – where the effects of parameters combine to influence the results. In some cases, the direct effect of parameters can be zero, while the interaction effects of those same parameters are significant. There are different categories of methods available to conduct GSA. These include derivative-, distributional- (of which variance-based are a sub-type), variogram-based- and regression-based-approaches ( Razavi *et al.*, 2021).

Variance-based sensitivity measures compute the proportion of the variance in the output that is explained by variation in the input. Computation of a first order (only the direct effect) and total-order (direct and interaction effects) index for each input recovers all interaction and non-linear effects of a model. However, variance-based sensitivity measures require a relatively large number of model runs; 500( *k*+2) runs, where k is the number of sensitivity parameters ( Saltelli *et al.*, 2008, 273).

An alternative to a variance-based approach is the derivative-based approach called elementary effects, or “Method of Morris” ( Morris *et al.*, 2008; Morris, 1991; Ruano *et al.*, 2012; Saltelli *et al.*, 2008), from here on referred to as Morris. This method reduces the number of model runs to 10( *k*+1), provides an estimate for the total-order index produced by more advanced variance-based approaches and can be applied to groups of inputs to further reduce computational requirements. However, the trade-off for this efficiency is that Morris will only provide information on influential and non-influential factors, as well as factors that have interaction and/or non-linear effects. This makes it suitable for factor prioritization and factor fixing, but not for understanding the nuances of how the factors interact with each other ( Campolongo *et al.*, 1999). A benefit of Morris (and some other GSA methods, such as Sobol) is the ability to group parameters to reduce the computational requirements. When you use parameter groups, Morris is appropriate for most ESOMs that have runtimes of at least 10 minutes with 100s of input parameters. Parameters allocated to groups all move together, but from different levels in the grid, so while the individual effects of the parameters within each group are no longer distinguishable, the global benefits of the GSA remain ( Saltelli *et al.*, 2008, 89–96).

Another alternative for performing a GSA on a large or very computationally demanding model is to use meta-modelling approaches. These use a statistical model, or emulator, trained to replicate the relationships between the model inputs and outputs. Interrogation of the parameters of the statistical model provides insights into the sensitivity of the model to input parameters ( Saltelli *et al.*, 2008, 212–35).

Choosing an appropriate GSA method is an exercise in trade-offs. Variance-based methods are computationally demanding for large models with many parameters, but give detailed insights, such as quantifying the interaction effects between combinations of inputs ( Saltelli *et al.*, 2008, 10–40). Morris provides limited insights into interaction effects, however, has a significantly lower computational cost than variance-based approaches ( Saltelli *et al.*, 2008, 109, 273). The accuracy of meta-modelling methods are dependent upon the quality of the meta-model and can be complex to implement effectively ( Borgonovo & Plischke, 2016).

Once the method is chosen, the analyst selects the range and distributions of the model parameters to be included in the analysis. The model is then run many times while the parameters are permuted to different values within their range. Some methods (such as Morris) require a specifically designed sample to be used, while others can be conducted using an arbitrary sampling approach such as Latin Hypercube or performed upon an existing ensemble of results. Finally, the sensitivity indices are calculated for each result variable by comparing the value of the input parameter with the change in the result variable.

### Benefits of global sensitivity analysis are attractive for ESOMs

In this section, we explore the aspects of GSA that are particularly attractive for ESOMs. We show that the primary benefit is that GSA helps a modeller to better understand the model’s behaviour, verify and validate the ESOM results, and develop consequential scenarios. Below we describe how GSA can be used to prioritise which parameters should be included in a scenario analysis (prioritisation), identify the aspects of the model which are redundant (fixing), and map interesting parts of the model output space to the corresponding region of the input space (mapping).

***Prioritise model inputs for your scenario analysis.*** By conducting a “factor prioritisation”, GSA can improve the quality of a scenario analysis by identifying the key inputs that influence the ESOM objective function (normally discounted total system cost), other decision variables (emissions, electricity price) or derived indicators (such as fraction of electricity generated from renewables) ( DeCarolis *et al.*, 2017, 192). Factor prioritization enables the analyst to be confident that all important parameters are included and reduce the risk of surprises, such as unexpected non-linear behaviour and interaction effects ( Saltelli *et al.*, 2008, 156).

Most GSA methods provide a measure of sensitivity or influence sufficient for factor prioritisation, but the measures that are provided by the various methods often need to be interpreted differently. Computationally intensive methods, such as Sobol`, quantify 1 ^st^ order (direct) effects, 2 ^nd^ order and higher interaction effects, when two or more parameters work together to influence the model results, as well as a total-sensitivity index, which includes direct and all higher order interaction effects ( Saltelli *et al.*, 2008, 156, 161–62). However, computationally cheaper methods such as Morris still provide a robust estimate of the influence of input parameters which is sufficient to measure the relative importance of model inputs ( Saltelli *et al.*, 2008, 111).

***Fix input parameters that are redundant.*** Conducting scenario analysis can be especially frustrating when time is spent carefully developing a new scenario, only for the results to be largely identical to those that exist. Global sensitivity analysis can help prevent this situation by identifying parameters which do not influence the results ( Saltelli *et al.*, 2008, 156). Factor fixing allows the analyst to proactively manage the issues of redundant data by reducing the dimensionality of an ESOM ( Saltelli *et al.*, 2008, 156). A systematic approach to managing the data intensity — the many hundreds or thousands of input parameters in ESOMs – is highly desirable to simplify model maintenance and upkeep, and to increase confidence in the model behaviour.

Computationally efficient methods such as Morris, are particularly useful for factor fixing as the sensitivity measure is robust ( Saltelli *et al.*, 2008, 125). If Morris determines that a parameter is uninfluential with a sensitivity index value close to 0, then it is unlikely to affect the model results and does not need to be the focus of further uncertainty investigation.

Together, factor fixing and factor prioritisation prevent the modeller from classifying a non-influential factor as influential and/or classifying an influential factor as non-influential (known as Type I and Type II errors respectively in GSA).

***Map interesting regions of the output space to input parameter ranges.*** A related use of GSA methods is identifying regions of the output space of interest, perhaps for policy development, and mapping those combinations of input parameter values required to recreate them. This method involves propagating a Monte Carlo sample through the model, and using statistical methods, such as the patient rule induction method (PRIM) ( Friedman & Fisher, 1999) and classification and regression trees (CART) ( *Classification And Regression Trees*, 2017) to identify areas of the model input space that are highly predictive of an area of interest in the model output.

### Advances in the availability of global sensitivity analysis methods

The availability of tools and methods to implement GSA has significantly improved in recent years. Several open-source software packages are available in some common programming language, including SALib ( Herman & Usher, 2017; Iwanaga *et al.*, 2022) for Python, GlobalSensitivity.jl ( Dixit & Rackauckas, 2022) for Julia, VARS-TOOL ( Razavi *et al.*, 2019), SAFE ( Noacco *et al.*, 2019) and UQLab for Matlab, and sensobol ( Puy *et al.*, 2022) for R. These range from providing a full graphical user interface for uncertainty quantification and GSA, through to robust implementations of various methods.

While these software packages make the sample creation and post-processing of results relatively easy, there are still several conceptual and practical hurdles to cross before completing a GSA for an ESOM – as well as how to balance computational demands with ease and depth of interpretability of the sensitivity analysis results. In this paper, we use Python and SALib to conduct GSA and compute the sensitivity indices of an ESOM.

## Literature applying GSA to ESOMs

As can be seen in Table 2, the list of papers that report the application of a GSA method to an ESOM-like model is relatively short, especially considering the growth in energy system optimisation studies over the same time period ^2^. One major issue is the poor recognition of the value GSAs provide ESOMs in the literature, even though GSA is listed in a highly cited paper on ESOM best-practices ( DeCarolis *et al.*, 2017). For example, a 2018 review ( Yue *et al.*, 2018) found just nine papers out of 2,100 uncertainty-related ESOM studies which propagate uncertainty through a model or perform a SA. The review presented an uncertainty technique flowchart but does not situate GSA within the available approaches (as distinct from Monte Carlo analysis).

**Table 2.** The limited number of sensitivity analysis studies performed on ESOMs (FF: factor fixing; FP: factor prioritisation; FM: factor mapping).

Paper	Model	Method	Setting	Number of Variables
( Campolongo & Braddock, 1999)	TIMER	( Morris, 1991)	FP	6
( van der Sluijs *et al.*, 2002)	TIMER	( Morris, 1991)	FF	300
( Van Der Sluijs *et al.*, 2005)	IMAGE, TIMER	( Morris, 1991)	FP	300
( McJeon *et al.*, 2011)	GCAM	full factorial	FP	99
( Alzbutas & Norvaisa, 2012)	MESSAGE	partial correlation coefficients	FP	6
( Scott *et al.*, 2014)	GCAM	fractional factorial	FP	5
( Anderson *et al.*, 2014)	DICE	( Plischke *et al.*, 2013)	FP	51
( Butler *et al.*, 2014)	DICE	( Sobol’, 1990)	FP	30
( Bosetti *et al.*, 2015)	WITCH, GCAM, MARKAL	DMIM ( Plischke *et al.*, 2013)	FP	8
( Branger *et al.*, 2015a)	Res-IRF	( Morris, 1991)	FF & FP	71
( Pye *et al.*, 2015)	ESME	Regression	FP	~106
( Moret *et al.*, 2016)	Swiss-EnergyScope	( Morris, 1991)	FP	600
( Usher, 2016)	ESME	( Morris, 1991)	FP	200
( Moret *et al.*, 2017)	Swiss-EnergyScope	( Morris, 1991)	FF & FP	372
( Moksnes *et al.*, 2019)	SAMBA	Scenario discovery	FM	6 groups
( Groves *et al.*, 2020)	OSeMOSYS-CR	Scenario discovery	FM	348
( Tröndle *et al.*, 2020)	Euro-Calliope	Sobol’	FP	12
( Yliruka *et al.*, 2022)	HIT	( Morris, 1991)	FP	20

Of the GSA studies in Table 2, a subset uses non-ESOM models and are limited in scope/detail. Although technically, the TIMER, IMAGE, GCAM and DICE models are not ESOMs, they share some similar characteristics and raise some useful insights. ( Campolongo & Braddock, 1999) uses the Method of Morris to explore the influence of six input parameters of three outputs of the IMAGE model, using a fixed ±10% range, noting the favourable trade-off of insight versus the increased computational burden of Morris (128 runs) over that of factorial approaches. A Morris analysis on TIMER ( van der Sluijs *et al.*, 2002), a component of the IMAGE model, was performed to reduce the dimensionality of the model. The authors note that the choice of arbitrary bounds ±50% may have biased the results. Two studies on GCAM ( McJeon *et al.*, 2011; Scott *et al.*, 2014) perform an analysis using full- and fractional-factorial approaches respectively. They note that many different combinations of technologies substitute for one another which reduces the influence of related parameters. This illustrates the inverse relationship between flexibility and sensitivity of a model (one interpretation of “robustness”).

Some of the studies identified apply useful techniques but are limited in their scope and/or have limited application. For example ( Bosetti *et al.*, 2015) compares the influence of eight technology attributes across three different models. The results illustrate structural differences between the models, but the findings are limited due to the small number of parameters explored.

Two studies apply GSA to ESOMs and provide some insights into the limitations and potential benefits of the approach. Moret *et al.* (2016) use Morris to explore the influence of 600 parameters of an ESOM varied around their central value. Despite not estimating uncertainty ranges for the inputs, the results show that relatively few input parameters drive the results, including energy service demands, technology efficiency and operating and investment costs. Moret *et al.* (2017) build upon this work by detailing a comprehensive approach that combines factor fixing and factor prioritisation on the same MIP model, in a two-stage approach using Morris. This study suggests an uncertainty classification method and uses GSA to highlight how selecting a fixed uncertainty range can lead to Type I and Type II errors.

A comprehensive analysis of a technologically detailed residential demand model conducted using Morris ( Branger *et al.*, 2015a) found that only a handful of parameters were responsible for most uncertainty in the outputs. Several useful insights for the interpretation of results from Morris are presented in the paper, including how to interpret non-linear and interaction effects, deal with missing data and how the arbitrary nature of quantifying the uncertainty of input parameters may influence the results.

Scenario discovery is an approach that implements the setting of “factor mapping”. Six categories of parameters were permutated to generate 324 distinct scenarios of the South American continent using the SAMBA model ( Moksnes *et al.*, 2019). The results were then clustered across five dimensions (such as CO _2_ emissions, investment costs as a percentage of gross domestic product (GDP), capital investment, stranded assets and fixed and variable costs) and the PRIM algorithm was used to identify the key determinants of the results. A similar approach was applied for Costa Rica ( Groves *et al.*, 2020), where 3,003 scenarios were mined using clustering and PRIM algorithms to identify “vulnerabilities” and their determinants.

( Tröndle *et al.*, 2020) analytically derive Sobol’ indices from a meta-model fitted to model results using polynomial chaos expansion (PCE) routines available in UQLab ( Marelli & Sudret, 2014). The analysis measures the influence of 12 technology cost parameters upon total system cost and the total capacity of five technology categories across three different modelling scales. The advantage of using PCE is that the surrogate model can be fit to the existing model runs generated using a Latin hypercube sampling approach, which is computationally cheaper than variance-based approaches.

( Yliruka *et al.*, 2022) presents a methodology to rank both the input parameters (such as energy demand) and modelling decisions (such as spatial resolution) together using GSA. They find that accounting for the uncertainty associated with model parameters and model decisions are dependent on the result variable of interest.

In summary, while there are a few isolated examples of good practice, in general, the implementations of GSA to ESOMs are limited, even among this subset of studies that have made the (considerable) effort to do so. The primary limitations of the studies listed are coverage of only a subset of input parameters with a predominant focus on technology costs, use of arbitrary ± *X*% ranges for uncertain inputs, and the computational demands of more advanced methods. Other issues raised include implementation details, such as how to translate sampled uncertainty ranges for input parameters into time series that can be used in the ESOM.

There are general insights that can be drawn from these analyses to inform future efforts. Moret’s detailed methodological description ( Moret *et al.*, 2017) suggests a two-stage approach that uses a computational cheap factor fixing method, such as Morris, to screen out uninfluential input parameters, followed by a more computationally expensive factor prioritisation to elicit more detailed information about interaction effects. The paper also offers a taxonomy of uncertainty that distinguishes between parameter types. Further, the paper confirms that the use of fixed ranges influences the sensitivity analysis results. Several of the papers ( Branger *et al.*, 2015a; Usher, 2016) confirm the finding echoed by ( Saltelli *et al.*, 2008), that most uncertainty in model outputs is influenced by a small number of input parameters, but tellingly, this is only shown (and can only be shown) in studies which include input parameters from different categories and do not only focus upon technology costs.

Given these findings, we list good practices for conducting a global sensitivity analysis of an ESOM to address the gaps identified in the literature:

- Include as many parameters as possible from different categories, and do not only explore technology costs
- Quantify ranges of uncertainty for each of the parameters rather than using arbitrary ranges
- Follow a two-stage process of factor fixing using Morris, followed by factor prioritisation using a more computationally demanding approach for fewer parameters.

## Methods

In this section we describe a sequence of ESOMs on which we later perform GSA, moving from the simplest possible example (Model 1) to one which exhibits many of the more realistic characteristics observed in current research settings (Model 3). In designing these examples, we intended to show a clear link between the intuitive behaviour you would expect from the ESOM and the results from the GSA. To facilitate the pedagogical aims of this paper, we start with a very simple example and then add complexity. We continue by explaining how we developed a GSA workflow and the features of the workflow. Finally, we conclude the section by describing the result indicators obtained from a GSA and how to interpret them in the context of an ESOM.

### Defining the sequence of models of increasing structural complexity

**Figure 1.**
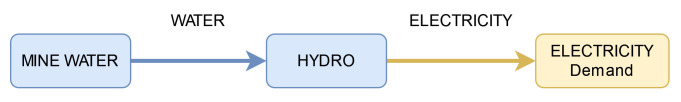
Energy model with a single dispatchable technology.

Model 1 is a simple energy model with a single dispatchable hydro powerplant and a single electricity demand, as shown in Figure 1. The hydro plant has a capital cost, a fixed cost, an efficiency, and an operational life, while the electricity demand grows at a consistent yearly rate. To ensure consistency across the models, we use a time horizon of 50 years, eight equal time slices per year, and a fixed discount rate of 5%. Model 1 has complete freedom to choose the optimal design of an energy system – investing in new capacity in the first year to meet demand for the remainder of the model horizon.

**Figure 2.**
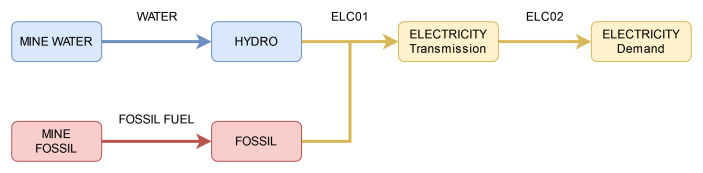
Energy model with multiple dispatchable energy technologies.

Model 2 investigates how competition of technologies effects the supply cost curve. As shown in Figure 2, we add a fossil fuel generation station that has similar parameters to the hydro station, in addition to variable costs (fuel) and a representative emission factor. More advanced model behaviour and policies can now be tested such as yearly capacity addition limits (build rates), carbon pricing and emission targets. Unlike the first model, Model 2 has existing “residual capacity" in the system, for which initial capital investment are not required; this will represent a system undergoing an energy transition.

**Figure 3.**
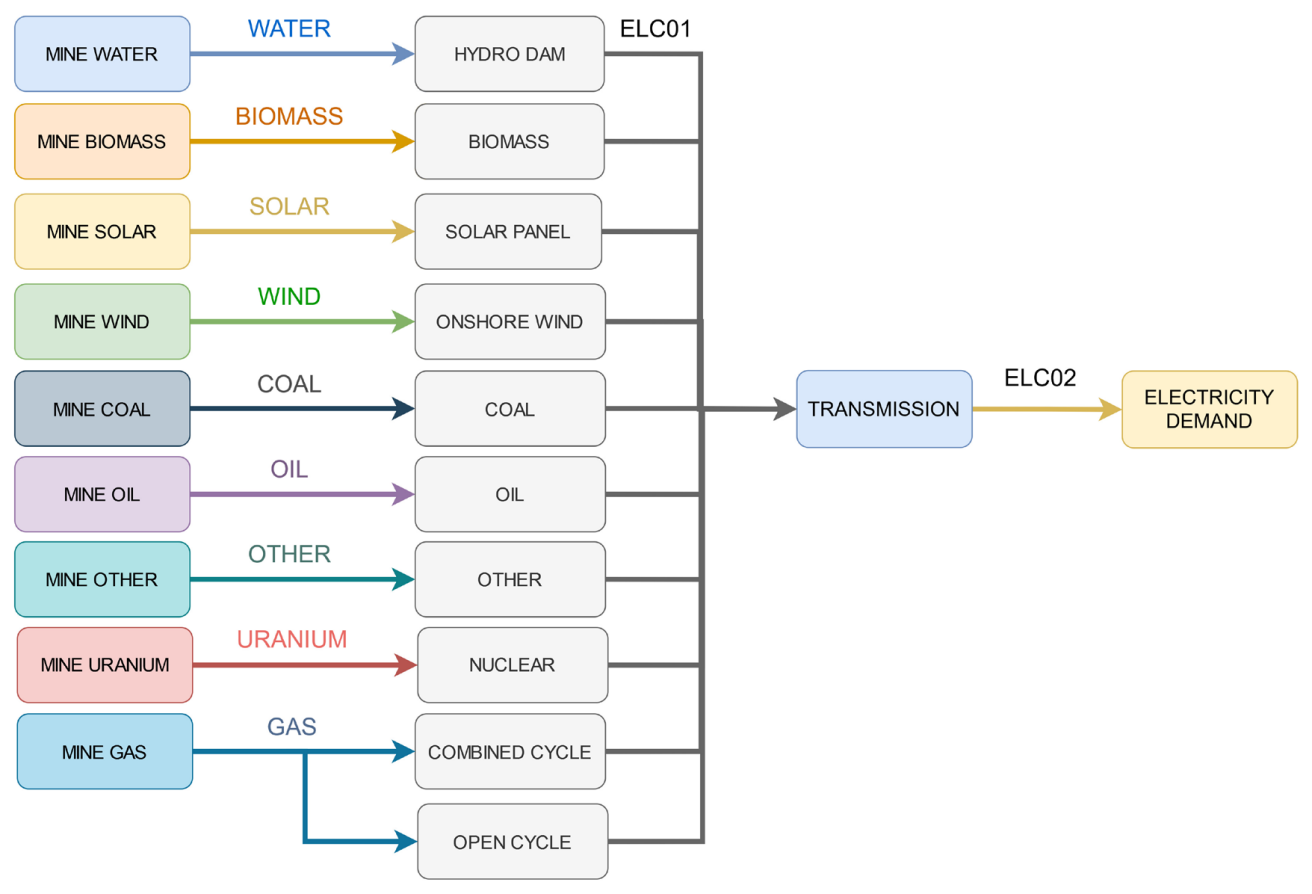
The reference energy system of the Nepal node in OSeMSOYS Global.

In Model 3, we use OSeMOSYS Global ( Barnes *et al.*, 2022) to generate a model for Nepal and demonstrate the behaviour under GSA of a typical ESOM, with many generation technologies representing alternative energy supply chains. We choose Nepal as it is represented by a single node in OSeMOSYS Global, thus, reducing the number of parameters to test. A simplified version of the reference energy system is shown in Figure 3. The “Other” technology refers to residual capacities from the system, added by OSeMOSYS Global, that don’t fall into a specific classification and cannot be invested in.

### Sample model results

**Figure 4.**
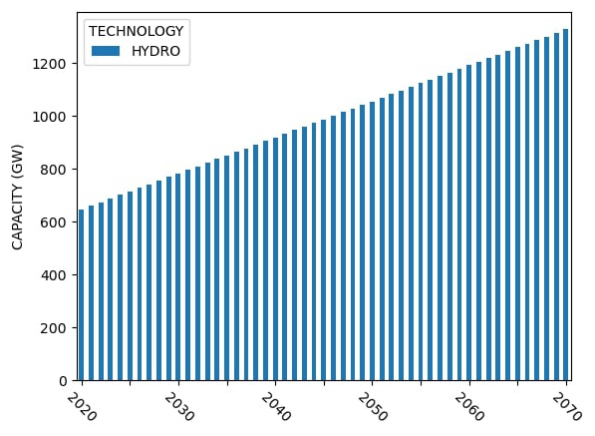
Model 1 installed capacity.

Sample capacity, generation and investment results for Model 1 are shown in Figure 4 – Figure 6.

**Figure 5.**
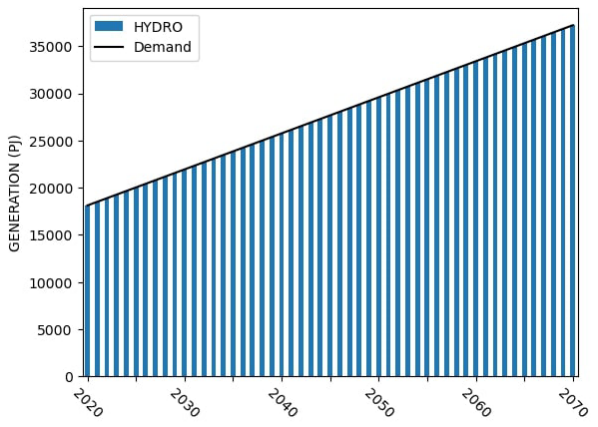
Model 1 electricity generation.

**Figure 6.**
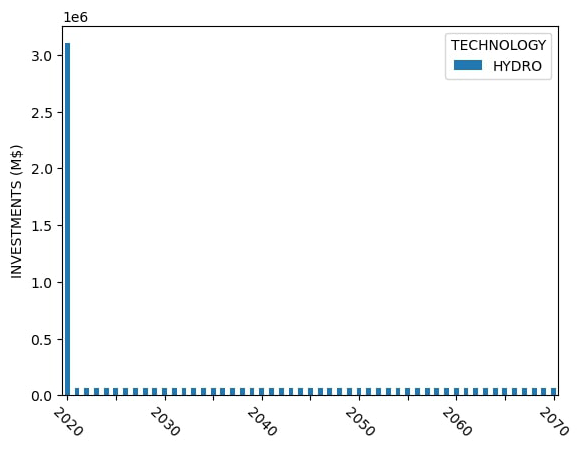
Model 1 capital investments.

**Figure 7.**
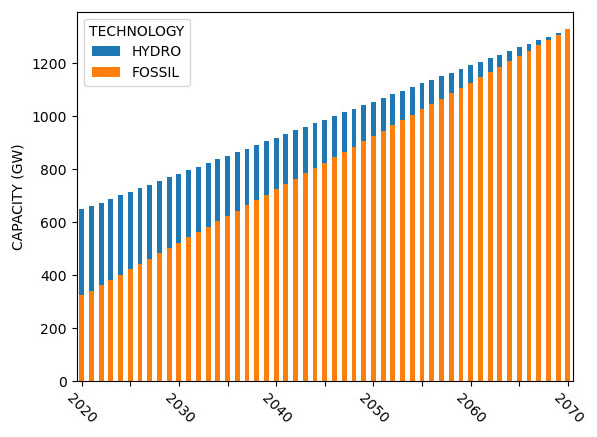
Model 2 installed capacity.

Sample capacity, generation, investment, and emission results for Model 2 are shown in Figure 7 – Figure 10.

**Figure 8.**
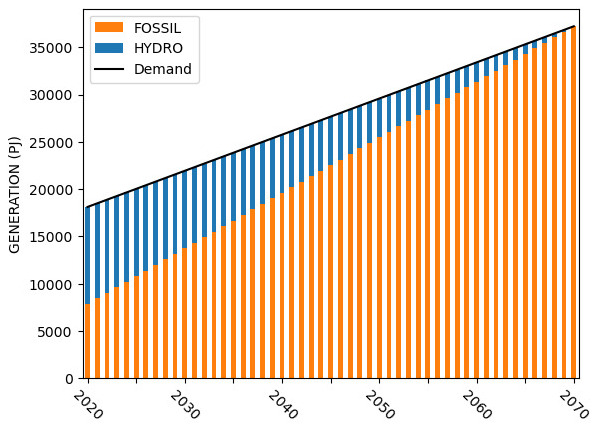
Model 2 electricity generation.

**Figure 9.**
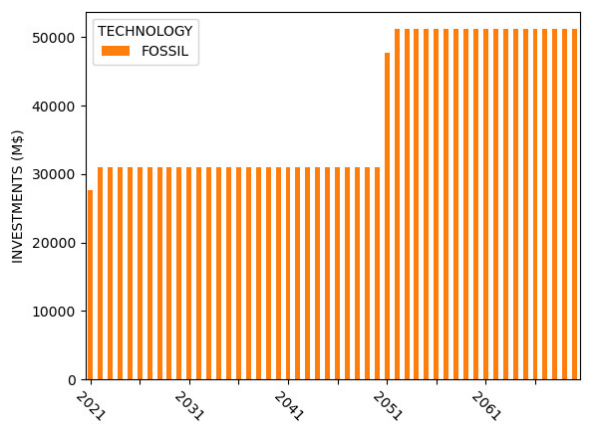
Model 2 capital investments.

**Figure 10.**
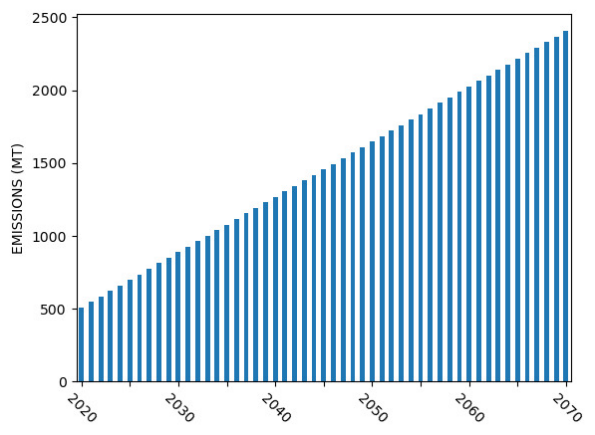
Model 2 emissions.

### Implementation of the global sensitivity analysis

**Figure 11.**
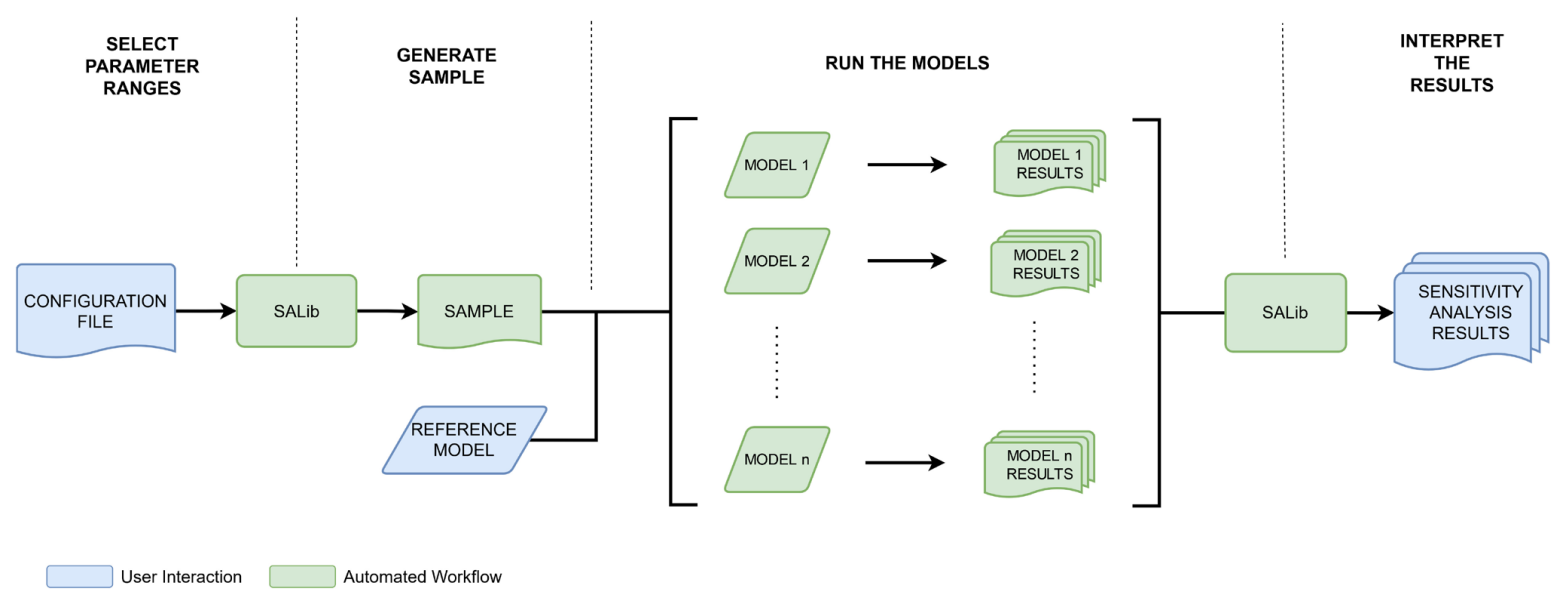
High level sensitivity analysis information flowchart.

To perform the GSA, we use the open-source SALib ( Herman & Usher, 2017; Iwanaga *et al.*, 2022) Python package (RRID:SCR_023074). The process of conducting the analysis is split into several steps. First, SALib is configured with information on the number, ranges, and distributions of the model parameters. SALib generates a sample using the appropriate sample function for the method chosen. Next, the samples are “expanded”. Each row in the sample corresponds to one model run, and the parameter values in each column of the row must be written into the model data file. Then, each of the model runs must be performed, and results extracted from each of the runs. Finally, the analyse function from SALib is used to calculate the sensitivity indices. This process is shown in Figure 11.

A Snakemake workflow is built and openly shared to conduct all GSA in this paper **.**. Snakemake ( Mölder *et al.*, 2021) (RRID:RRID:SCR_003475) is a Python-based workflow management tool that meets these GSA configuration requirements and can be scaled to easily execute workflows on high performance computers. The workflow is responsible for reading in user configuration files, sequentially calling scripts to generate the SALib (RRID:SCR_023074) sample, creating and solving the many different models, and displaying the result graphs and tables. Snakemake will build and solve each individual model in parallel (the computationally intensive portion of the workflow) since model runs are independent. This drastically reduces workflow execution time. This workflow can be used as is for any GNU MathProg implemented version of OSeMOSYS, or it can be modified and/or replicated for any ESOM.

### Selecting parameter ranges

While SALib simplifies the sample creation process, it does not replace the need for the modeller to select sensible input values. Input values will include quantified uncertainty ranges over which the parameters will be varied; arbitrary uncertainty ranges can easily bias the results. Various sources of uncertainty can be explored in a sensitivity analysis of an ESOM. Technology parameters may be uncertain due to our lack of knowledge about the future (epistemic uncertainty), or due to natural randomness (aleatory uncertainty). Analysts may use values from literature, results from other models or expert judgement to provide plausible ranges that can be used. As this is a pedagogical focused paper on GSA, we use uncertainty ranges that clearly highlight the effects of GSA. Table 3 and Table 4 shows the ranges used in the Model 1 and Model 2 examples respectively, while the ranges used for Model 3 (the OSeMOSYS Global run) can be found in Table 5. All parameters follow uniform distributions, however, in practice the distribution will be dependent on the specific parameter and model. It should be noted that while we do not explore the effect of spatial and temporal resolution to simplify the working examples, in practice these dimensions are also important to explore.

**Table 3.** Sample parameter ranges used in Model 1.

Parameter	Min Value	Max Value
Capital costs hydro	2000 *M$⁄GW*	5500 *M$⁄GW*
Fixed costs hydro	30 *M$⁄GW*	60 *M$⁄GW*
Demand	18000 *PJ*	56000 *PJ*

**Table 4.** Sample parameter ranges used in Model 2.

Parameter	Min Value	Max Value
Capital costs hydro	2000 *M$⁄GW*	5500 *M$⁄GW*
Fixed costs hydro	30 *M$⁄GW*	60 *M$⁄GW*
Capital costs fossil	500 *M$⁄GW*	2000 *M$⁄GW*
Fixed costs fossil	30 *M$⁄GW*	60 *M$⁄GW*
Variable costs fossil	0.5 *M$⁄PJ*	3 *M$⁄PJ*
Fossil efficiency	25%	50%
Operational life hydro	50 years	100 years
Operational life fossil	30 years	60 years
Demand	18000 *PJ*	56000 *PJ*
Emission limit	18500 *MT*	37000 *MT*

**Table 5.** Uncertainty ranges for Model 3’s global sensitivity analysis.

Parameter	Min Value	Max Value	Unit
Capital Cost Biomass	1680	2100	*M$⁄GW*
Capital Cost CC Gas	632	790	*M$⁄GW*
Capital Cost Coal	1152.8	1441	*M$⁄GW*
Capital Cost Hydro	1655.2	2069	*M$⁄GW*
Capital Cost OC Gas	340	425	*M$⁄GW*
Capital Cost Oil	992	1240	*M$⁄GW*
Capital Cost Solar	893.6	1117	*M$⁄GW*
Capital Cost Nuclear	2612	3265	*M$⁄GW*
Capital Cost Onshore Wind	1268	1585	*M$⁄GW*
Fixed Cost Biomass	59.0	88.5	*M$⁄GW*
Fixed Cost CC Gas	21.0	31.5	*M$⁄GW*
Fixed Cost Coal	40.0	60.0	*M$⁄GW*
Fixed Cost Hydro	41.0	61.5	*M$⁄GW*
Fixed Cost OC Gas	17.0	25.5	*M$⁄GW*
Fixed Cost Oil	16.8	25.2	*M$⁄GW*
Fixed Cost Solar	14.0	21.0	*M$⁄GW*
Fixed Cost Nuclear	129.0	193.5	*M$⁄GW*
Fixed Cost Onshore Wind	22.8	34.3	*M$⁄GW*
Variable Cost Coal	1.4	2.2	*M$⁄PJ*
Variable Cost Gas	3.0	6.7	*M$⁄PJ*
Variable Cost Oil	5.8	11.6	*M$⁄PJ*
Variable Cost Uranium	4.0	6.0	*M$⁄PJ*
Efficiency Biomass	27.3	34.1	%
Efficiency CC Gas	50.0	62.5	%
Efficiency Coal	34.3	42.8	%
Efficiency OC Gas	35.1	43.9	%
Efficiency Oil	32.6	40.7	%
Efficiency Nuclear	92.6	99.0	%
Operational Life Biomass	36	54	*years*
Operational Life CC Gas	26	36	*years*
Operational Life Coal	26	36	*years*
Operational Life Hydro	80	100	*years*
Operational Life OC Gas	26	36	*years*
Operational Life Oil	26	36	*years*
Operational Life Solar	26	36	*years*
Operational Life Nuclear	58	72	*years*
Operational Life Onshore Wind	26	36	*years*
Demand	18.94	122.08	*PJ*
Carbon Tax	20	100	*M$/MT*
Max Coal Capacity	0.5	4.0	*GW*

### Generating samples

When conducting a GSA, a sample is generated which contains information needed to modify the input parameters and create the array of models to runs. In the workflow, SALib is configured with information on the number, ranges, and distributions of the model parameters. SALib generates a sample using the prescribed user inputs and GSA method selected.

**Figure 12.**
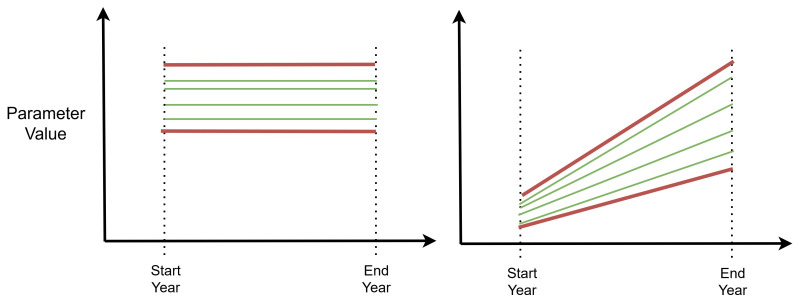
Model parameter configuration options for ( **a**) time-independent parameters, ( **b**) time-dependent parameters with a fixed start point and moving end point. Red lines represent user inputs, while the green and red lines represent sampled values.

The next step is to transform the scalar sampled values into timeseries for the respective ESOM parameters. Normally, an ESOM will include a base (or historical) year for which values are known, and sensitivity analysis is not needed. Following the base year, parameter uncertainty can be time-independent or time-dependent. Time-independent parameters, as shown in Figure 12a, do not change throughout the model horizon, such as a technologies operational life or the discount rate. Time-dependent parameters include factors that vary over the model horizon, such as demand or fuel costs, as shown in Figure 12b.

In the workflow we developed ( Usher *et al.,* 2023), we statically define the parameter configuration options so that the intermediate values can be interpolated when creating the model runs from the sample. Table 6 shows how a time-dependent parameter is configured, in this case for the capital cost of nuclear. Four values are given to define the ranges at the start year and end year. The sample value from SALib, is actually provided as a value between 0 and 1, which is then multiplied to provide the linearly interpolated values for each of the model years between start year and end year.

**Table 6.** Configuration of a time-dependent parameter in the workflow.

parameter	group	indexes	start year		end year	
			min value	max value	min value	max value
CapitalCost	capital	"R1,NUCLEAR"	4000	5000	2000	3000

### Run the models

Once the sample is generated, the corresponding models are created and run. Due to the data intensive nature of ESOMs, computation time for a single model run can easily be hours long. Exacerbated by the fact that GSA methods scale according to the number of parameter (or parameter groups), data management and computational requirements can quickly become overwhelming for large models with many parameters ( Usher *et al.,* 2023a). Therefore, implementing a workflow, such as the Snakemake workflow described, to manage the data handling and parallelized model runs is beneficial.

The model creation and solving process involves converting the mathematical representation of the system into a generalized LP data file, calling a solver to solve the model, and saving the model results. Our workflow uses the Python package otoole ( Usher *et al.*, 2021) for conversion of OSeMOSYS data files, and the GNU Linear Programming Kit ( ‘GLPK - GNU Project - Free Software Foundation (FSF)’ n.d.) to generate the model file. We can then call the open-source solver CBC ( Forrest *et al.*, 2022), or the commercial solvers CPLEX or Gurobi. Results from these runs are saved in CSV format, which are later compiled and used as inputs for SALib.

Caution must be taken when selecting input parameter ranges, because a modeller can easily create a situation where a model run is infeasible. For example, if an aggressive emission limit is sampled in conjunction with strict build limits on renewable technologies, the model may not be able to meet demand within the prescribed conditions. In these cases, the workflow will flag an error and the modeller will need to revisit the uncertainty ranges attached to the input parameters.

### Interpret the results

Morris produces three sensitivity measures *μ*, μ* and *σ*, all of which are summarized in Table 7. Equation (1) – Equation (5) highlight how to calculate the elementary effect, and how to calculate the sensitivity indicators described in the Method of Morris ( Morris, 1991; Morris *et al.*, 2008; Ruano *et al.*, 2012; Saltelli *et al.*, 2008). When calculating the elementary effect, Equation (1) is used if the *i ^th^* component of *x* ^( *l*)^ is increased by Δ, else Equation (2) is used if the *i ^th^* component of *x* ^( *l*)^ is decreased by Δ. See ( Saltelli *et al.*, 2008), chapter 3 for more information on the formulation of the Method of Morris.

**Table 7.** SALib Method of Morris sensitivity results.

Parameter	Description
*μ*	Provides the mean of the elementary effects with respect to a result parameter. The higher the value, the more influential is the parameter. It can be positive or negative and indicates the direction of the effect on the model output but can be susceptible to errors if the effects are both positive and negative. Not available when groups of parameters are used. Units correspond to the result parameter.
μ*	Provides the mean of the absolute elementary effects. The value is always positive and cannot indicate the direction of the effect on the model output but is a more reliable measure of influence. The higher the value, the more influential the parameter. Units correspond to the result parameter.
*σ*	The standard deviation. Provides an indication of the nonlinear effects and/or interactions with other factors. Not available when groups of parameters are used. Units correspond to the result parameter.
μ* Confidence Interval	Provides the 95% bootstrapped confidence interval for μ* values. The standard deviation cannot be calculated when using parameter groups, therefore, SALib will provide a confidence interval for μ* which can be used as a proxy for the standard deviation. Units correspond to the result parameter.

$$EE_{i}^{j}(x^{\left(l\right)})=\frac{\left[y(x^{\left(l+1\right)})-y(x^{\left(l\right)})\right]}{\Delta }\;(1)$$

$$EE_{i}^{j}(x^{\left(l+1\right)})=\frac{\left[y(x^{\left(l\right)})-y(x^{\left(l+1\right)})\right]}{\Delta }\;(2)$$

$$\mu_{i}=\frac{1}{r}\sum_{j=1}^{r}EE_{i}^{j}\;(3)$$

$$\mu_{i}^{*}=\frac{1}{r}\sum_{j=1}^{r}\left|EE_{i}^{j}\right|\;(4)$$

$$\sigma_{i}^{2}=\frac{1}{r-1}\sum_{j=1}^{r}{\left(EE_{i}^{j}-\mu \right)}^{2}\;(5)$$

Where:

$EE_{i}^{j}$ *= The Elementary Effect of the factor i on the j ^th^ trajectory*

*r* = *Number of Trajectories*

*l* = *Trajectory*

*i* = *Input Factor*

Δ = *Step Size*.

Morris computes an average of absolute local derivatives (or *elementary effect*) for each parameter while all other inputs are moved over the gridded space (defined by a step size) of input parameter ranges. Each set of movements is called a *trajectory*. Each trajectory consists of *k*+1 movements, where *k* is the number of input parameters or parameter groups. For each of the analyses presented in the results section, Morris was configured to run with a step size of 4 and generate a sample of 10 optimal trajectories from a pool of 100 randomly generated trajectories.

As noted earlier, performing a GSA on an ESOM allows the modeller to analyse the entire suite of result variables, such as costs, capacity investments, and emissions. However, μ* (the measure of influence) is given in units of the result variable, such as USD, capacity, energy, or weight. Therefore, to compare the influence of multiple factors with different units on one graph we can express μ* as a percentage of $\mu_{\max}^{*}$ for each result variable.

A note should also be made on how to interpret interaction and non-linear effects ( *σ*) in the context of ESOMs. Interaction effects are when "factors intensify, cancel, or compensate for the effects of each other” ( Razavi & Gupta, 2015). For example, consider a single technology system (such as Model 1) that includes two sensitivity parameters, fuel costs and efficiency. The variable operating cost may be defined following Equation 1, where the ESOM will solve for the decision variable *operation*.

Variableoperatingcosts=(efficiency)×(fuelcosts)×(operation)(6)

The fuel costs and efficiency of the technology interact as summarised in Table 8. If the fuel costs rise or the efficiency decreases, we expect system cost to increase. However, if both increase or decrease, a portion of the changes will cancel one another out. A higher efficiency system will use less fuel, but if the fuel is more expensive, the cost savings will not be realised.

**Table 8.** Description of interaction effects between fuel costs and efficiency for a single technology energy model.

Fuel costs	Efficiency	Total costs	Type of interaction
Increase	Decrease	Increase	Amplify
Increase	Increase	Increase or decrease	Cancel or compensate
Decrease	Decrease	Increase or decrease	Cancel or compensate
Decrease	Increase	Decrease	Amplify

## Results

In this section we present GSA results using each of the three models described in the methodology section. We begin with a demonstration applying GSA to Model 1 to prioritise input parameters with respect to the objective function – discounted total system cost. Using Model 2, we then show how GSA can be used to compare the influence of both policy and technical parameters and then extend the analysis to include additional result variables beyond discounted total system cost. Finally, we demonstrate the application of GSA to the more realistic Model 3. This provides an opportunity to demonstrate how GSA can identify non-influential parameters that can be fixed to reduce the dimensionality of the model.

Given the pedagogical aims of the paper, our discussion is interwoven with the results. The discussion interprets and highlights how the analyst can use the results. The quantitative results are displayed on horizontal bar charts that are generated using SALib. The magnitude of the bar shows the μ* value, while the black line overlaid on the bar shows the μ* 95% confidence interval.

### Measuring the influence of parameters and avoiding Type 1 errors

**Figure 13.**
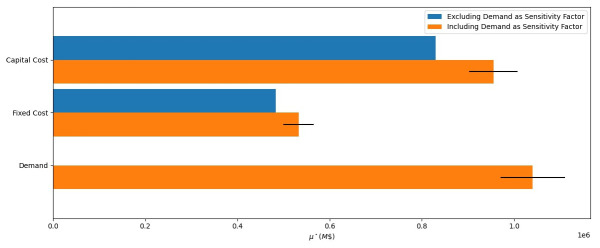
Global sensitivity analysis results for model one. Run one only includes capital costs and fixed costs in the sensitivity analysis. Run two add demand to the sensitivity analysis, highlighting it as a more influential factor. The magnitude of the bars shows the influence of the input parameters with respect to discounted total energy system cost.

Model 1 has three uncertain parameters: capital costs, fixed costs, and yearly electricity demand (see Table 3). In Figure 13 we show the results of two GSAs of Model 1, one of which omits a key uncertain parameter. In both analyses, capital costs are more influential than fixed costs, with μ* values of $0.83M and $0.96M compared to $0.48M and $0.53M respectively. Furthermore, in the run that holds demand at a central value, rather than varying it, no interaction effects are seen; signalling the model will respond linearly under linear changes to either capital costs or fixed costs.

More importantly, Figure 13 highlights the Type I error experienced by excluding demand from the analysis; we miss identifying it not only as an influential parameter but *the* most influential parameter at $1.04M. The addition of demand also changes the relative influence of capital costs and fixed costs. While their prioritization order didn’t change in this situation, models with many parameters can easily show a change in prioritization order. This demonstrates that when conducting a GSA, all uncertain parameters need to be included. This will accurately rank the influence of each parameter (factor prioritization) by taking all other uncertain parameters into account and ensuring we capture the most influential factors in the model.

Adding demand into the sensitivity analysis also introduces interaction effects, as shown by the overlaid black lines in Figure 13. Since we use Morris, we cannot draw conclusions on which parameters are interacting with one another, only that interaction effects exist ^3^. However, given the small number of parameters, we can hypothesise that as demand increases, the size of the supply system must increase, and therefore the range of capital costs means that there is an amplification effect. The interaction effects are therefore between demand and capital cost, and demand and fixed cost.

This simple example demonstrates the need to perform SA on all parameters in the model. Excluding demand as a sensitivity factor resulted in a Type I error, where we failed to identify an influential factor. Conducting a GSA gives us a method to rank the influence of parameters (factor prioritization) to develop effective scenarios (discussed further in the “Synthesis: Conducting a GSA of a more realistic ESOM” section).

### Efficiently verifying model behaviour by comparing technical and policy parameters

The introduction of competing energy technologies and policy interventions into an ESOM further emphasizes the benefits of conducting a GSA. Due to the rational decision-making assumption in ESOMs (see Table 1), technology investments will often exhibit “penny-switching” behaviour. The cost minimisation objective means the model will exclusively invest in the cheapest technology until a trade-off point is reached, at which point it will exclusively invest in a competing technology. Policy interventions, such as emission limits, emission taxes, subsidies, limits on technology capacity, capacity growth rates, renewable energy portfolio standards or investment limits, can be implemented as constraints or otherwise parameterised by the modeller to better represent reality. GSA helps increase our understanding of how these policies influence the model results relative to other model parameters. In Model 2, we increase the number of uncertain parameters in the model (see Table 4).

**Figure 14.**
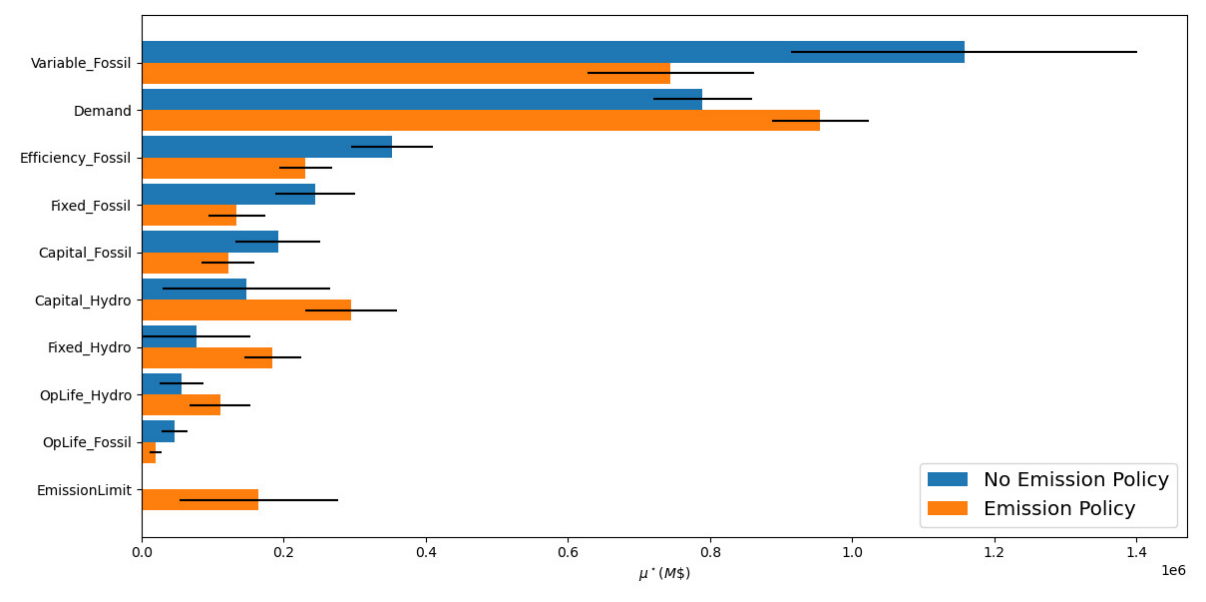
Global sensitivity analysis results for Model 2, a model with competing technologies. Blue bars represent a model with no emission limit, while orange bars represent the same model with an emission limit. The magnitude of the bars shows the influence of the parameters with respect to discounted total energy system cost.

In Figure 14 we show GSA results from Model 2 with an uncertain emission limit policy that constrains emissions to 25–50% of the optimal system emissions (this range is pre-calculated using results without the emission policy).

Comparing the results with and without the emission policy highlight the need to perform GSA on not only uncertain parameters, but also uncertain policies. For example, the capital costs of hydro stations jump from the sixth to the third most influential parameter after implementing the emission policy ( μ* = $0.147M to $0.295M). This further highlights the need to include all uncertain parameters that could be varied in the model in a GSA to avoid Type I and Type II errors.

Including uncertain policies in our GSA also provides us with a method to verify and validate the functionality of our model. Intuitively, we can reason out the changes seen by the emission policy in Figure 14. The emission limit will cap the amount of generation from fossil stations and force expensive hydro stations to be built, thereby, increasing the impact hydro stations will have on system level costs. In this capacity, GSA can act as a troubleshooting tool to help the analysist understand and calibrate the model.

Interaction effects are also affected by the addition of the emission limit. For example, looking at the variable fuel costs, the emission policy causes the mean influence of this parameter to decrease from M$1.157 to M$0.744 along with the μ* confidence interval (M$0.243 to M$0.117), and thus interaction effects. This decrease in interaction effects is caused by the emission policy reducing the space of possible outcomes as the other parameters change. As the emission limit becomes stricter, the amount of fossil fuel generation must go down and hydro generation must go up.

Imposing an emission policy upon an ESOM will fundamentally change the structure of the energy system represented. This system change is not (or may not) be well characterised by the result variable used so far: discounted total system cost. To best capture the range of effects that a parameter can have, it is important to explore the sensitivity of all relevant result variables to the inputs. Below we explore expanding the analysis to other result variables to demonstrate how further insights can be generated with negligible extra computational cost.

### Measuring influence across different result parameters

To capture the range of effects that an input parameter can have, it is important to explore the sensitivity of all relevant result variables to the inputs. The relative influence of the input parameters may differ considerably across result variables, and individual result variables do not capture the full range of insights from the model. For example, in addition to the objective cost, an ESOM will report results for technology capacity, technology operation, emissions, and investments, operation and fuel costs. It can be informative to generate indicators that better align with the insights required of the model which combine various result dimensions into one measure, such as “percentage of electricity generated by renewable technologies” and then calculate the influence of parameters against this. It is therefore important to consider which result variables are relevant when reporting results from a GSA. In general, these should align with the insights that are sought from the model.

**Figure 15.**
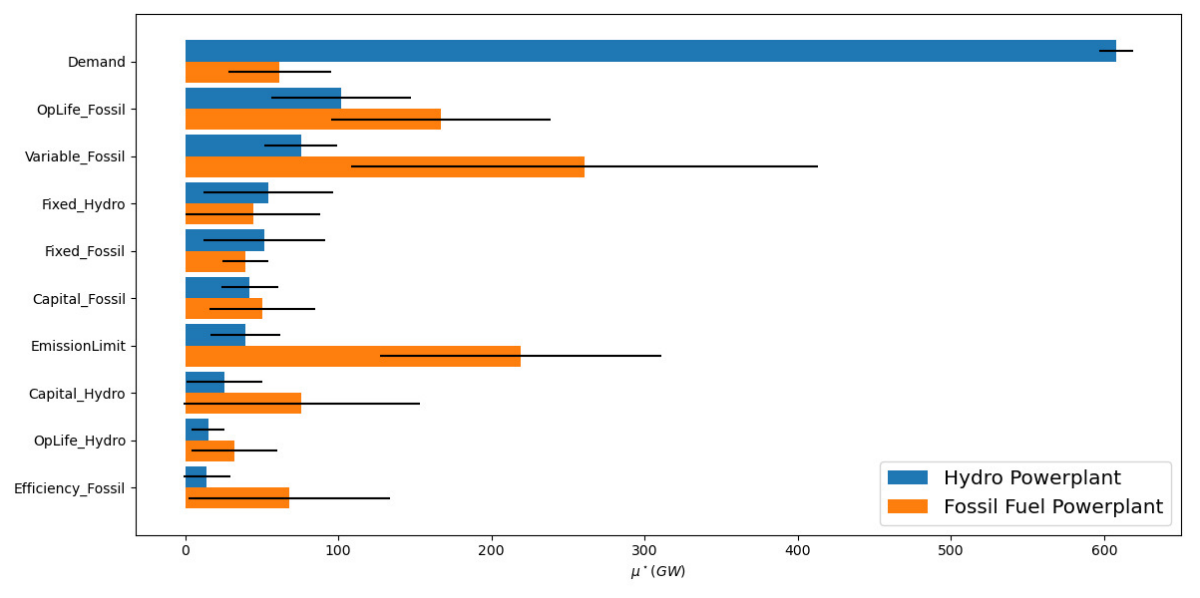
Results comparing the global sensitivity of total new capacity by technology in Model 2.

Once the model runs have been generated, such analyses can be conducted at negligible computational cost, as the GSA routine is computationally cheap to perform with the main effort burden lying with the building and solving of each ESOM. For example, we use the same Model 2 runs with the emission policy and conduct a GSA on total new capacity by technology in Figure 15. Immediately noticeable is how the factor prioritization order changes between the result variables investigated – total new capacity of hydro (blue) versus total new capacity of fossil fuel (orange). Demand is the most significant driver of hydro powerplant capacity investment (607GW), while variable fossil fuel costs (260GW), the operational life of fossil fuel stations (167GW), and the emission limit (219GW) are the drivers of fossil fuel capacity investments. Moreover, these factor prioritization orders are different than those seen for the objective cost.

Identifying the drivers of different model results can help confirm model behaviour and shape scenario analysis given the specific research question. For example, we know that at the nominal values the model prefers to install fossil fuels (see Figure 9), therefore, we should expect the emission limit to significantly impact the installed capacity. This behaviour is validated in Figure 15 by the emission limit having a large influence on fossil fuel capacity (219GW). Moreover, one might be quick to think emission limit will also have a large impact on hydro station capacity, yet demand is the main driver (607GW compared to 39GW). This behaviour intuitively makes sense though. We are restricting the amount of fossil fuel in all models runs, with the remaining generation capacity being taken up by hydro. Therefore, varying demand should have a significant effect on hydro capacity. Identifying this type of model behaviour results are especially impactful if scenario analysis is aiming to investigate one of these result variables. While the objective cost is often a key metric to look at, this single metric will abstract out the complexities of how the model is functioning and can lead to misguided scenario analysis.

**Figure 16.**
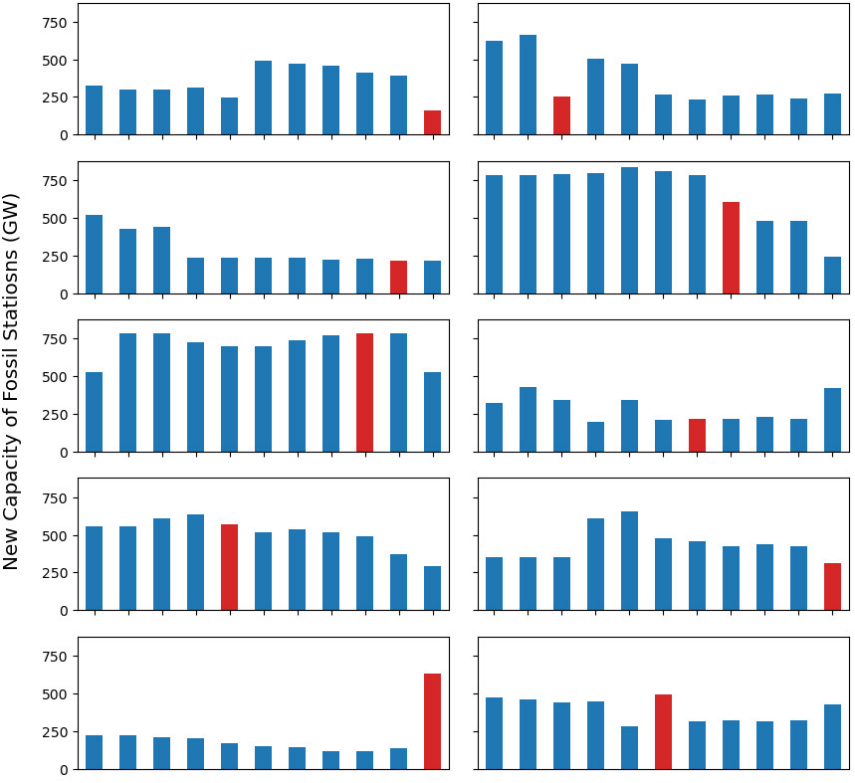
Model 2 with the emission policy results for new fossil station capacity for all model runs. The red bar indicates when variable fuel cost change with the sample. Often this change is accompanied by a step in the installed capacity, signalling that there will be interaction effects.

Figure 15 also shows significant interaction effects, which is partially caused by technological competition. Due to the rational decision-making assumption in ESOMs, in the absence of any constraints, in each run the model will exclusively invest in whichever is the cheapest technology, a phenomenon termed penny switching. This effect is exaggerated in this simple model with just two competing technologies. For example, variable fossil fuel costs and the emission limit have a large interaction effect with the new capacity of fossil fuel powerplants, at 152GW and 100GW respectively. This will likely signify that in some scenarios the variable cost is a binding constraint on hydro investment, and in others not. See Figure 16 for a graphical representation of this.

A challenge that arises from using multiple result variables is the difficulty in assessing the relative influence of parameters across result variables. When using Morris, the sensitivity measure is presented in the unit of the result variable, and it is left to the analyst to make the judgement across result variables. We explore one strategy to manage this in the next section where GSA is applied to a realistic ESOM.

### Synthesis: conducting a GSA of a more realistic ESOM

**Figure 17.**
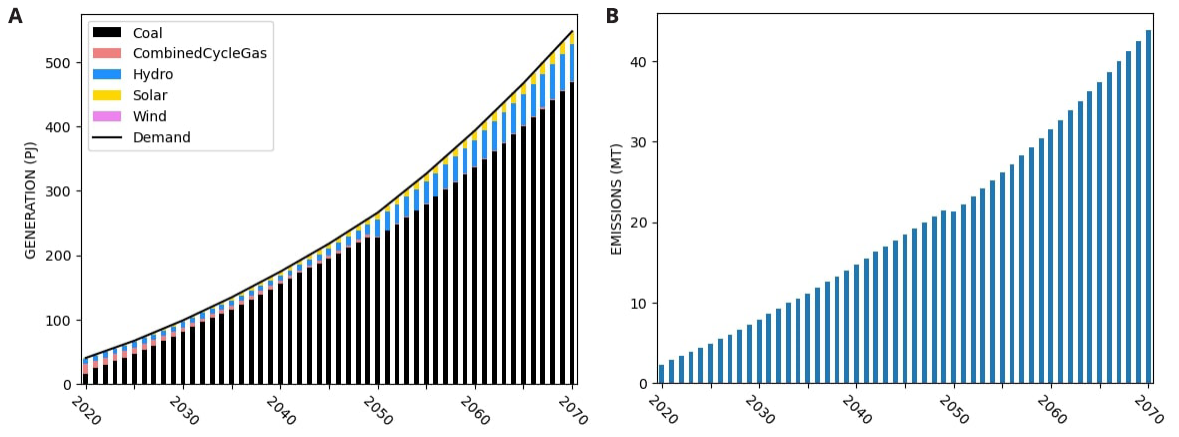
Nominal results from OSeMOSYS Global Run for Nepal for ( **a**) system generation and ( **b**) yearly emissions

In this section, we present the results of applying the GSA concepts discussed above to a realistic ESOM. To start, we develop a simplified research question; “What is the least cost mix of technologies that could help Nepal reduce its CO _2_ emissions to zero by 2070?”. We first run the model at our nominal values to understand the system. The generation and emission results are show in Figure 17. Immediately noticeable is the significant build out of coal power plants and increasing emissions.

**Figure 18.**
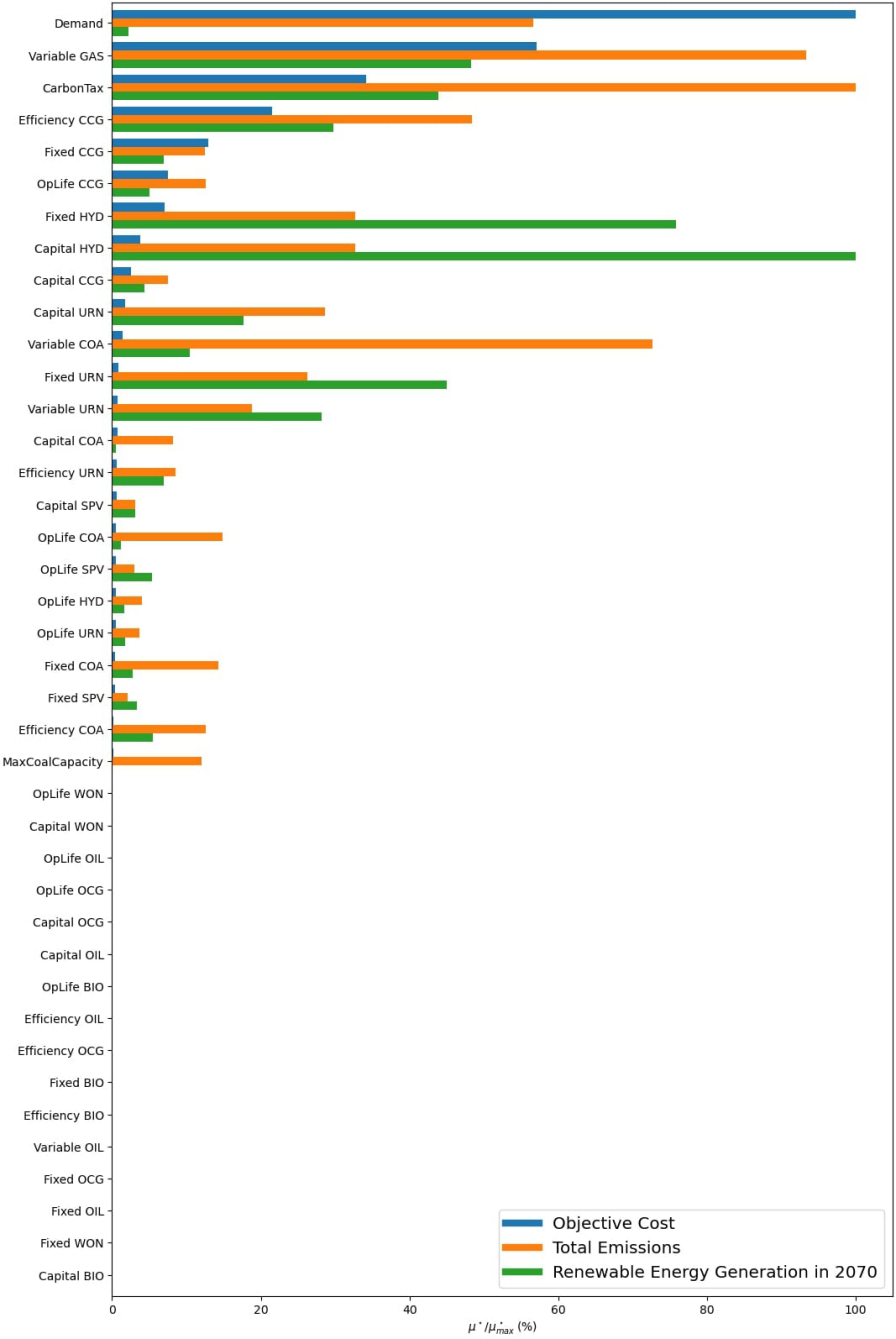
GSA Results using a Nepal model built by OSeMOSYS Global. The μ* values are presented as a percentage of the $\mu_{\max}^{*}$ for that result variable, allowing results with different units to be explored in one graph.

**Figure 19.**
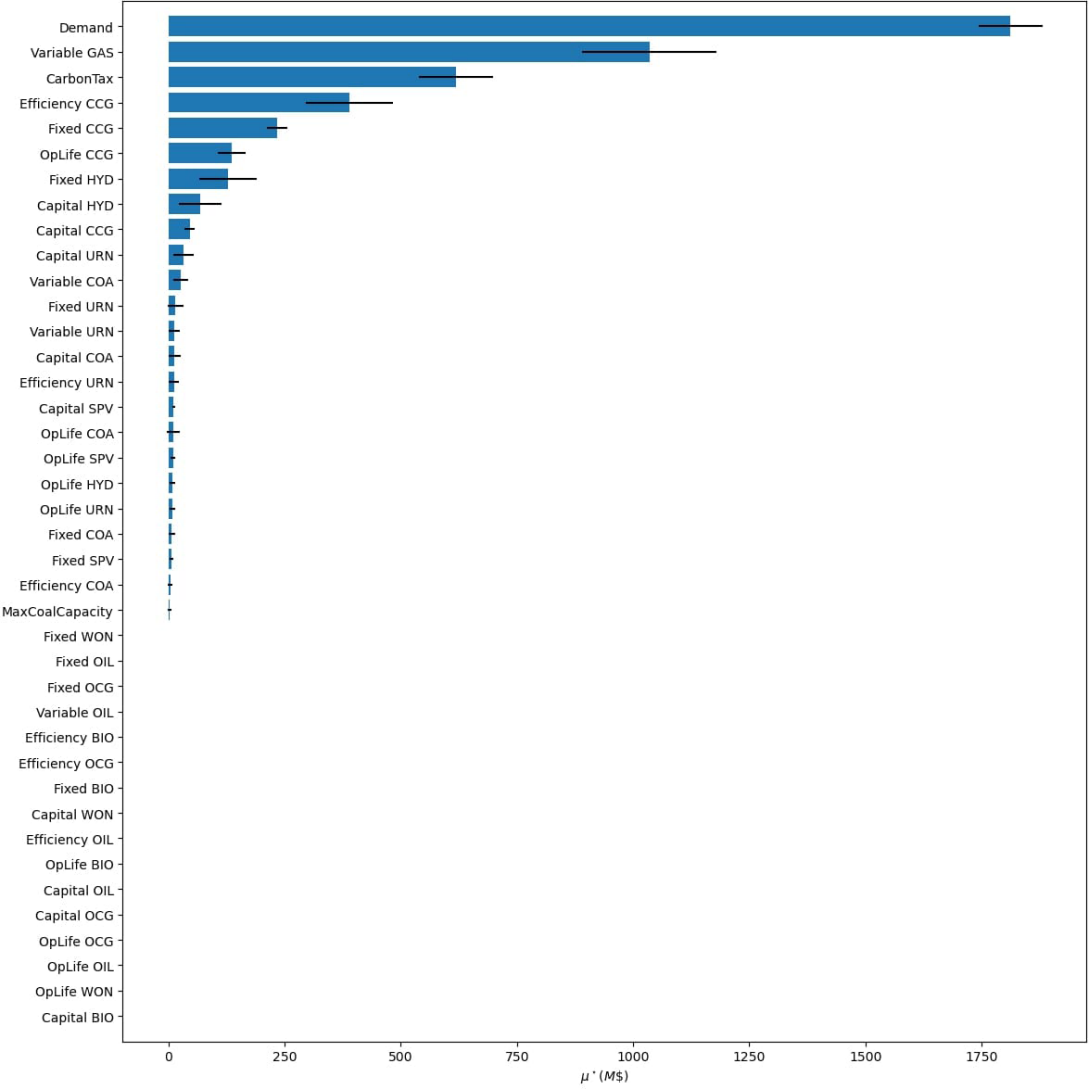
Objective cost GSA results from Model 3.

Next, we want to develop scenarios to help answer our research question with the help of a GSA. We first assign uncertainties to all parameters, such as costs, efficiencies, and demand. Moreover, we add in a carbon tax and build limits on coal as uncertain policies. For simplicity, we do not allow trade with surrounding nations, assume no energy storage options, and do not test the sensitivity of our temporal settings, reserve margin, and discount rate. The results of the GSA are presented in Figure 18, where we explore the objective cost, the model period emissions, and the percentage of renewable energy generation in 2070. To see individual result graphs, which include the interaction effects, see Figure 19– Figure 21.

**Figure 20.**
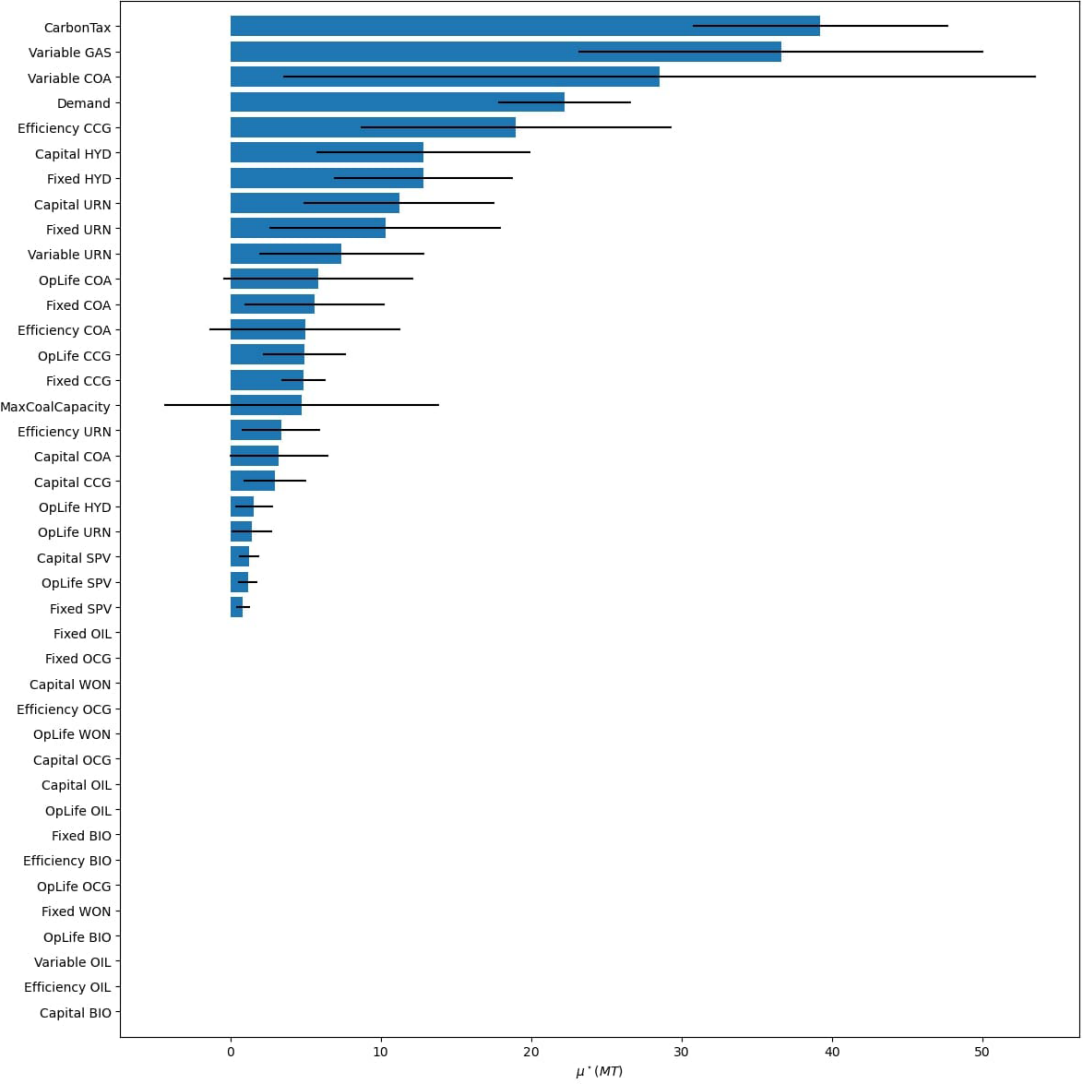
Model period emission GSA results from Model 3.

**Figure 21.**
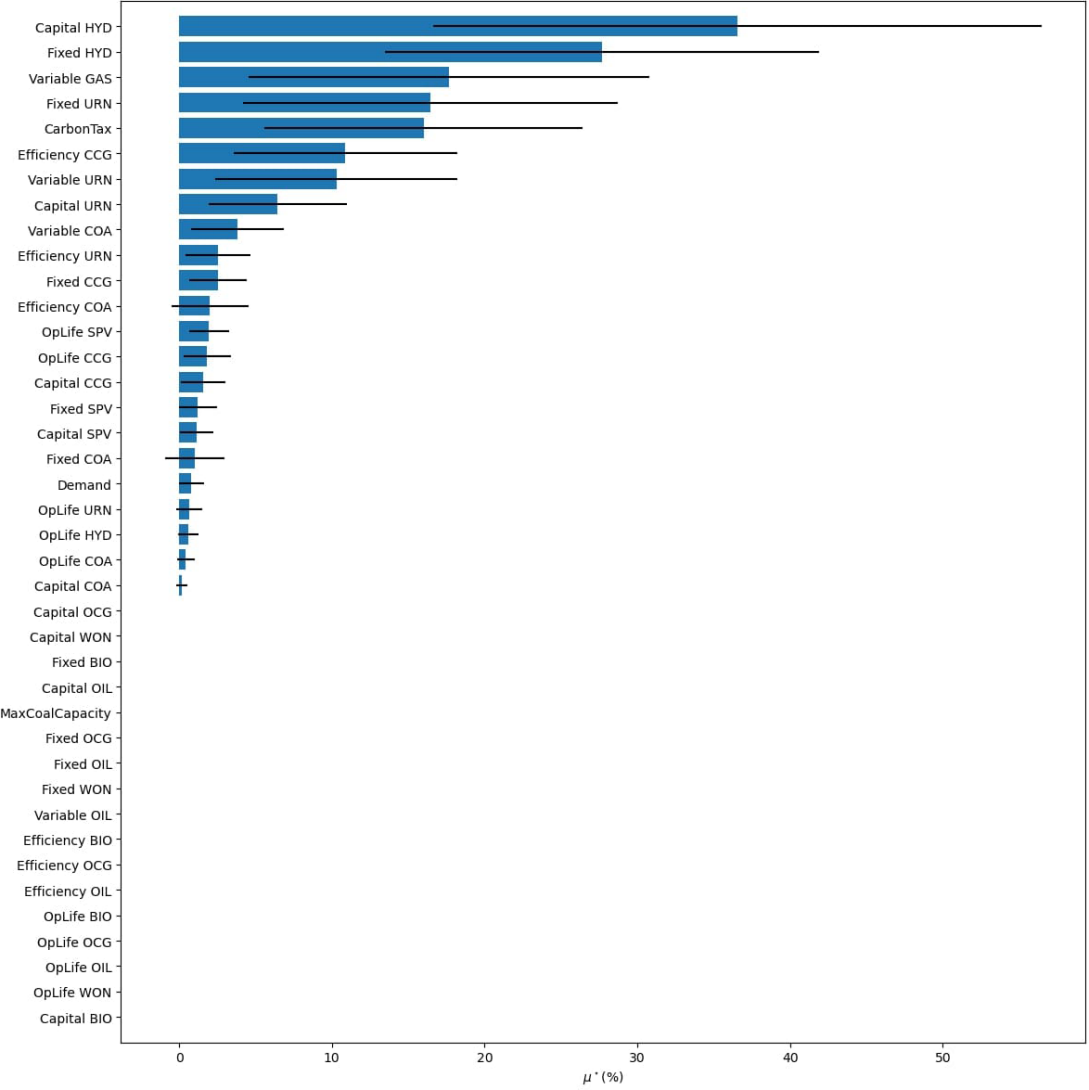
Renewable energy generation share GSA results from Model 3.

The GSA reveals that only a handful of factors significantly influence our results, the ranking of these factors changes depending on the result of interest, and that many parameters have minimal to no influence on one or more of the result variables. We can use these facts to build effective scenarios and verify that our model is behaving as expected.

First, we analyse the most influential factors in the model by result variable and tabulate these results in Table 9. This allows us to see common factors affecting our results of interest, and factors that only affect a subset. Once the factor prioritization of the inputs has been identified for the result variables deemed important, the modeller can use this information to build scenarios. Immediately noticeable is that both carbon tax and the variable costs of gas are present in all lists. Scenarios investigating these parameters will translate to consequential discussion points as the model will produce significantly different results. For example, subsequent scenario analysis which iterates through different variable gas costs and carbon penalties in a factorial design will produce noticeable changes in all the results of interest. Next, looking at objective costs in Figure 18, we see that demand (100% at M$1811) is substantially more influential than the next factor, variable gas costs (57% at M$1035). Therefore, it will be beneficial to include a scenario investigating different electrification trends as this will have a significant effect on system costs. With that said, we see that increased electrification scenarios will have a negligible impact on renewable energy generation penetration in 2070 (2.2% at 0.81PJ). Therefore, scenarios exploring the capital and fixed costs of hydro will provide a better understanding of renewable energy generation potential in 2070 (with μ* values of 30.4PJ and 25.1PJ respectively) .

**Table 9.** Factor prioritization order of Nepal’s global sensitivity analysis results, taken from Figure 10, where an influence of 1 is the highest μ* for each result variable.

Influence rank	Objective cost	Emissions	Renewable generation 2070
**1**	Demand	Carbon tax	Capital cost hydro
**2**	Variable cost gas	Variable cost gas	Fixed cost hydro
**3**	Carbon tax	Variable cost coal	Carbon tax
**4**	Efficiency CCG	Demand	Fixed cost nuclear
**5**	Fixed CCG	Efficiency CCG	Variable cost gas

**Figure 22.**
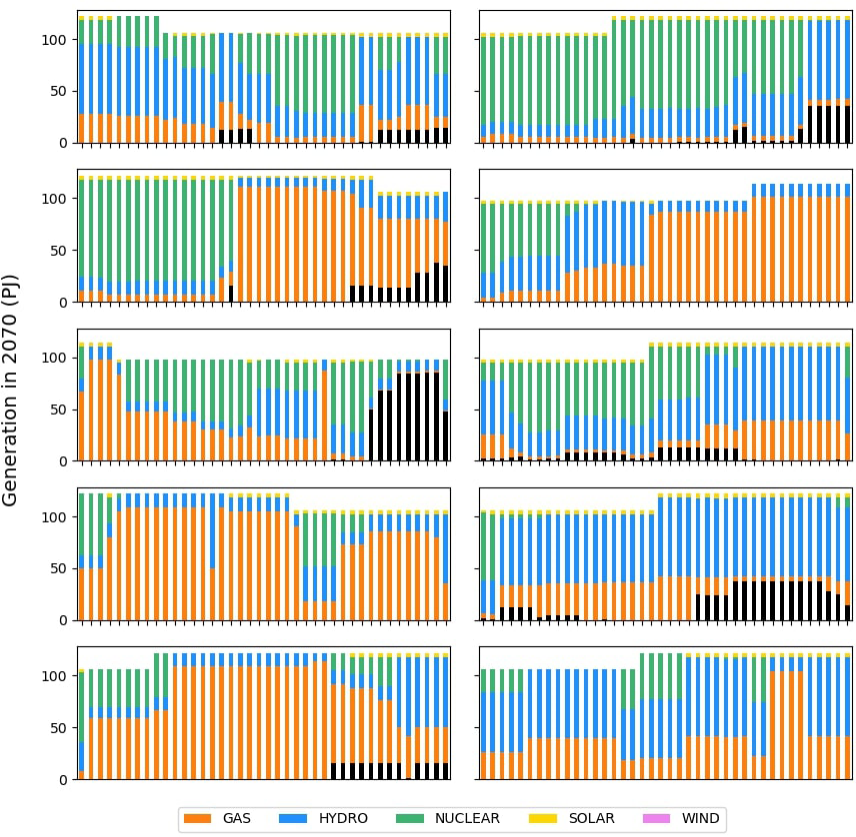
Method of Morris results for renewable energy generation share in 2070, based on Model 3.

Using a GSA to identify factor prioritization also helps in understanding the inner workings of the model. For example, demand has an almost negligible impact on the share of renewable energy generation in 2070 (0.81PJ). This may be surprising, but delving into the results will show that the generation mix is primarily dictated by other factors, such as the capital costs of hydro (30.4PJ). Simply changing the demand will increase or decrease the amount of generation from the different sources proportionally. For a graphical representation of this behaviour, see Figure 22. This shows how GSA is an efficient and comprehensive approach to understand the inner workings of the model within the set uncertainty ranges.

### Fixing non-influential parameters to reduce model dimensionality

The results shown in Figure 18 also demonstrate how factors can be fixed to reduce model dimensionality. The bottom 16 parameters in Figure 18 have a zero, or near zero, influence on any of the result variables. This means that whatever value this parameter is within its uncertainty range (see Table 5), it will not affect the objective cost, emissions, or renewable energy generation share in 2070, given the uncertainty ranges of all other parameters. This is a key finding as it allows us to easily make model behaviour conclusions to reduce the size of the model (removing uninfluential parameters), and save time by not investigating these parameters in a scenario.

For example, in Figure 18, all cost parameters relating to biomass have negligible influence when looking at any of the results. Therefore, we can confidently state that the model will not invest in or use biomass under the conditions presented within our uncertainty range. The alternative time-consuming and inefficient trial-and-error approach would be to guess at what conditions the system will invest in biomass, for example, low capital costs of biomass and high capital costs of all other technologies and check these conditions one by one.

We can go further and use these quickly obtainable conclusions to safely reduce the dimensionality of the scenario analysis or subsequent, more detailed, sensitivity analysis. As previously discussed, when performing scenario analysis simply based on experience or a few sample model runs, this can easily lead to Type I and Type II errors. Running a GSA and quantitively identifying what factors will not affect the results of interest will prevent these errors. This leads to scenario analyses where the analyst can be more confident in what exact factors are driving model changes. Moreover, if a second sensitivity analysis is conducted to, for example, extract higher-order interaction effects from the model, a more computationally expensive algorithm can be run, such as Sobol, that excludes these parameters. In summary, factor fixing provides the analyst with a systematic way to identify which factors do not influence model results and remove them from further analysis saving time and effort.

## Discussion

In this pedagogical paper, we demonstrate the benefits of GSA to ESOM analysts and suggest solutions to challenges associated with the GSA approach.

We showed, using a very simple ESOM, the importance of including all variables in a GSA to avoid Type I errors. The results from the GSA provide an absolute and relative rank of the influence of the parameters and an indication of interaction effects. Using a two-technology ESOM, we compare the results of the GSA with and without the imposition of a varying emission constraint. These results clearly demonstrate how the pattern of influential and non-influential parameters differ under these two policy settings and how the penny-switching behaviour emerges in the GSA results. As far as we know, no previous study has attempted to provide a clear interpretation of GSA results for ESOM analysts. Our finding for the more realistic model of Nepal which highlights the dominance of a minority of parameters is also shown for different models in ( Branger *et al.*, 2015b; Moret *et al.*, 2016). We also demonstrate that different result variables are sensitive to different patterns of input parameters. It is therefore important that the modeller ensures that they perform the GSA for result variables that inform key insights, and not only total costs and emissions.

Analysts can make use of these results in various ways. In practice, it is desirable for ESOMs to produce useful results when they are operated over a wide range of parameter values. GSA results can help efficiently verify the behaviour of the ESOM by identifying influential and non-influential parameters over the full range of the input space. The analyst can use the results of the GSA to design scenarios that include influential variables as key drivers or dimensions of a scenario analysis and can confidently exclude parameters that are identified as non-influential. In other words, our findings show that without GSA, analysts are left to guess at how influential one parameter is compared to another, which can easily lead to Type I and Type II errors when designing an ESOM scenario analysis.

While the benefits of GSA have been described, the challenges and limitations of this family of methods warrant discussion. ESOMs can often have long running times (in the order of multiple hours). This poses a significant issue, as performing GSA requires running the model many times. To overcome this, the modeller can use a two-step approach (as suggested by Moret *et al.*, 2017)) – first a “screening” method, using a more computationally efficient method, such as Morris used in this paper, followed by a more comprehensive approach if required. While Morris provides limited information compared to the more computationally expensive approaches, it still allows for the identification of influential, non-influential, and interacting parameters. We agree with the pioneering users of Morris with energy optimisation models (see Table 2), that these attributes mean that Morris is sufficiently powerful for use with ESOMs to supporting scenario analysis. A second challenge concerns the implementation of the sampling methods with model parameters. We described a process for interpolating the timeseries required for ESOM parameters which draw on good practices from the literature, but the implications of the assumptions should investigated further.

We also offer some personal reflections on the process of conducting a GSA of an ESOM. We have found that GSA provides a structured and more systematic approach to developing a scenario analysis using an ESOM. Without using GSA, the process of selecting parameters to include in a scenario analysis is largely done by experience, or modeller judgement, or worse trial and error without a formal understanding of the influence of those parameters upon the model output. With GSA, we arrive at clear conclusions faster and have more time to explore other avenues of interest. Quantifying the uncertainty around parameters and using these uncertainty ranges to conduct a GSA is a very useful exercise to understand the behaviour of the ESOM. Without GSA, one can probe the model to see if it is working correctly, but there is always a lingering doubt as to whether it will perform effectively under all conditions. With GSA, you more thoroughly explore the model, both the extremes and the interactions between parameters. This provides more confidence in the model's behaviour.

We use ESOMs to address issues that sit within the realm of post-normal science – large uncertainties and high stakes. Now is the time for GSA to become “ *an integral part of*” energy systems optimisation modelling. GSA minimises the risks associated with the alternative of ignoring uncertainty, and the consequent potential for misplaced confidence in results that could result in energy transition pathways that could lock us into a dangerous, sub-optimal or expensive future.

## List of abbreviations

**Table:** 

Abbreviation	Descriptions
ESOM	Energy System Optimisation Model
SA	Sensitivity Analysis
GSA	Global Sensitivity Analysis
LP	Linear Program
OSeMOSYS	Open Source energy Modelling System
GDP	Gross Domestic Product
MIP	Mixed Integer Program
CCS	Carbon Capture and Sequestration/Storage

## Data Availability

Zenodo: Global sensitivity analysis to enhance the transparency and rigour of energy system optimisation modelling - Supplementary Material. https://doi.org/10.5281/zenodo.7553059 ( Usher *et al.,* 2023a) This project contains the following folders of data: **Figures/** (This folder contains author-created graphics presented in the paper. The file Diagrams.drawio.xml is included, which can be imported to diagrams.net to view the source of these graphics.) **Model_One/** (Model one is a single technology OSeMOSYS model.) **Model/** (Contains the OSeMOSYS model, and model inputs and outputs as CSVs.) **Images/** (Images relating to model 1. These include capacity expansion result figures and GSA figures) **GSA/No_Demand/** (GSA configuration files are required to run Model 1 **without** the Specified Annual Demand as a sensitivity parameter.) **GSA/With_Demand/** (GSA configuration files are required to run Model 1 **with** the Specified Annual Demand as a sensitivity parameter. **model_one.ipynb** (Jupyter Notebook to generate all images shown in the Images/directory) **Model_Two/** (Model two is a multi-technology OSeMOSYS model.) **Model/** (Contains the OSeMOSYS model, and model inputs and outputs as CSVs. Two sets of model data are stored in this folder, one which does not have a ModelPeriodEmissionLimit, and one that does.) **Images/** (Images relating to model 2.) **GSA/No_Emission_Limit/** (GSA configuration files are required to run Model 2 **without** the Emission Limit.) **GSA/With_Demand/** (GSA configuration files are required to run Model 2 **with** the Emission Limit.) **model_two.ipynb** (Jupyter Notebook that generates all images shown in the Images/directory) **Model_Three/** (Model three is a multi-technology OSeMOSYS model of Nepal, generated by OSeMOSYS Global.) **Model/** (Contains the OSeMOSYS model, and model inputs and outputs as CSVs.) **Model/osemosys_global.yaml** (The configuration file used to generate results from OSeMOSYS Global.) **Images/** (Images relating to model 3.) **GSA/**(GSA configuration files are required to run Model 3.) **model_three.ipynb** (Jupyter Notebook that generates all images shown in the Images/ directory) Data are available under the terms of the Creative Commons Attribution 4.0 International license (CC-BY 4.0).

## Footnotes

^1^ The portfolio of technology options in an energy system optimisation model creates a piecewise linear supply-cost curve. This overall shape of this curve is normally a non-linear convex function. This can result in unexpected outcomes when ESOMs are run under extreme conditions, as the extremities of the supply curve are explored.

^2^ A SCOPUS search TITLE-ABS-KEY (energy AND system AND model AND optimi*ation ) AND ( LIMIT-TO ( DOCTYPE , "ar" ) ) AND ( LIMIT-TO ( EXACTKEYWORD , "Optimization" ) ) AND ( LIMIT-TO ( LANGUAGE , "English" ) ) returns 15,816 articles and shows an increase in publications from 12 papers per year in 1990 to almost 2000 per year in 2022.

^3^ If a more accurate picture of 2 ^nd^ and higher order interactions are needed, then a variance-based approach such as Sobol can be used.

## Notes

1. Only the summary results from the GSA results are included. Since the GSA will generate a large amount of data, viewing the complete suite of GSA results is left up to the user through running the workflow with the supplied data.
2. A copy of the OSeMOSYS model is included in each directory for consistency only. All three models use the same version of OSeMOSYS.
3. Each model has a datapackage.json and config.yaml file included in its input data. These are consistent throughout the models and are used for data processing with otoole

## Software availability

**Source code available from:** https://github.com/KTH-dESA/esom_gsa

**Archived source code at time of publication:** https://doi.org/10.5281/zenodo.7548829 ( Usher *et al.,* 2023)

**License: MIT**
