# Supplementary material for: Global sensitivity analysis to enhance the transparency and rigour of energy system optimisation modelling
Source: Open Res Eur. 2023 Feb 13;3:30. [Version 1] doi: 10.12688/openreseurope.15461.1 (PMC10445882; doi:10.12688/openreseurope.15461.1)
Supplement: Peer review history

# Peer review: Reviewer response for version 1

**DOI:** 10.21956/openreseurope.16716.r32995

**Published:** 2023-07-21

**Authors:** Neha Patankar

**Affiliations:** 1 Binghamton University, Binghamton, New York, USA

The paper titled " Global sensitivity analysis to enhance the transparency and rigour of energy system optimisation modelling" presents a comprehensive analysis of the application of the method of Morris, a one-at-a-time global sensitivity analysis technique, on energy system optimization models (ESOMs). The authors showcase the use of this technique through three test systems of increasing complexity, providing valuable insights into the uncertainty analysis of ESOMs.

 Following are minor comments for the authors:

1. Temoa, an open-source energy system model very similar to OSeMOSYS, has already implemented the method of Morris with its code published on GitHub. https://github.com/TemoaProject/temoa/tree/energysystem/tools/method_of_morris  
2. The following two papers have demonstrated the usage of the method of Morris in the ESOM contexts.
   
  1. Eshraghi, H., de Queiroz, A. R., & DeCarolis, J. F. (2018). US energy-related greenhouse gas emissions in the absence of federal climate policy. *Environmental science & technology*, *52*(17), 9595-9604. – This paper extensively described the parameter selection, sampling and method of morris procedures and demonstrates the utility of the method on a US test system.
  2. Patankar, N., de Queiroz, A. R., DeCarolis, J. F., Bazilian, M. D., & Chattopadhyay, D. (2019). Building conflict uncertainty into electricity planning: A South Sudan case study. *Energy for Sustainable Development*, *49*, 53-64. – This paper also extensively described the parameter selection, sampling and method of morris procedures and demonstrates the utility of the method on a South Sudan test system
3. To strengthen the pedagogical contribution of the paper, the authors should provide a detailed description of the methodology employed in these papers and explain how their study expands upon the existing literature.
4. The authors touch upon the issue of spatial and temporal correlation in parameters, such as natural gas prices, which necessitates consideration in ESOMs. While they mention the grouping issue, further elaboration on best practices for handling such cases would be valuable to the readers.  
5. Additionally, the authors highlight the arbitrary bounds on uncertain model parameters utilized in previous studies. However, they do not justify their rationale for determining uncertainty bounds or provide best practices. It would greatly enhance the paper if the authors refer to the framework established by the following two articles for determining uncertainty bounds and offer their insights on the best practices in this regard.
   
  1. Patankar, N., Eshraghi, H., de Queiroz, A. R., & DeCarolis, J. F. (2022). Using robust optimization to inform US deep decarbonization planning. *Energy Strategy Reviews*, *42*, 100892.
  2. Moret, S., Babonneau, F., Bierlaire, M., & Maréchal, F. (2020). Decision support for strategic energy planning: A robust optimization framework. *European Journal of Operational Research*, *280*(2), 539-554.
6. The authors discuss the use of snakemake files and the creation of a more efficient workflow. Given that the method of Morris runs, or individual trajectories, can be solved independently, incorporating commentary on parallel computing would provide practical guidance for researchers seeking to optimize computational efficiency.  
7. Although the authors employ uniform distribution for their analysis, it is worth noting that in practice, the triangle distribution has been more effective for trajectory creation in the method of Morris (Eshraghi et al. (2018)). Including a discussion on this observation would enrich the paper's recommendations for future studies.
8. Regarding the generation of samples, the authors could reference the works of Eshraghi et al. (2018) and Patankar et al. (2019), who utilized Latin hypercube sampling for method of Morris analysis. By explaining their selection criterion and visualizing the sampled trajectory for Model 1 on a two-dimensional grid, the authors can offer a helpful visual aid for building an intuitive understanding of the elementary effect.  
9. An important aspect that was not explicitly mentioned in the paper is that the method of Morris is a one-at-a-time global sensitivity analysis, meaning that only one uncertain parameter is changed at a time during a single model run. This characteristic aids in determining the elementary effect, and it would be beneficial for the authors to highlight this aspect for clarity.
10. The authors briefly touch upon the issue of infeasible model instances arising from overly wide uncertainty ranges. In practice, introducing a penalty factor prevents such cases. Expanding upon this idea would be beneficial, providing readers with a potential solution to handle infeasible scenarios.
11. The paper does an excellent job of addressing the interactions between uncertain parameters and the effects of broad policies on these interactions. Given the growing need for uncertainty analysis in the energy modeling community, this analysis is crucial, and the authors' contribution in this area is highly appreciated.

In conclusion, " Global sensitivity analysis to enhance the transparency and rigour of energy system optimisation modelling " offers a well-written and informative analysis that meets the rising demand for uncertainty analysis in the energy modeling field. The paper could be further improved by providing more comprehensive citations and explanations, highlighting practical recommendations, and clarifying certain aspects of the method employed. Overall, it is a valuable contribution to the scientific literature and serves as a useful resource for researchers in the field.

Is the study design appropriate and does the work have academic merit?

Partly

Is the work clearly and accurately presented and does it cite the current literature?

Partly

If applicable, is the statistical analysis and its interpretation appropriate?

Yes

Are all the source data underlying the results available to ensure full reproducibility?

Yes

Are the conclusions drawn adequately supported by the results?

Yes

Are sufficient details of methods and analysis provided to allow replication by others?

Yes

Reviewer Expertise:

uncertainty analysis in energy system optimization models

I confirm that I have read this submission and believe that I have an appropriate level of expertise to confirm that it is of an acceptable scientific standard, however I have significant reservations, as outlined above.

## References

[1] : US Energy-Related Greenhouse Gas Emissions in the Absence of Federal Climate Policy. *Environ Sci Technol* .2018;52(17) : 10.1021/acs.est.8b01586 9595-9604 10.1021/acs.est.8b01586 30129749

[2] : Building conflict uncertainty into electricity planning: A South Sudan case study. *Energy for Sustainable Development* .2019;49: 10.1016/j.esd.2019.01.003 53-64 10.1016/j.esd.2019.01.003

[3] : Using robust optimization to inform US deep decarbonization planning. *Energy Strategy Reviews* .2022;42: 10.1016/j.esr.2022.100892 10.1016/j.esr.2022.100892

[4] : Decision support for strategic energy planning: A robust optimization framework. *European Journal of Operational Research* .2020;280(2) : 10.1016/j.ejor.2019.06.015 539-554 10.1016/j.ejor.2019.06.015

# Peer review: Reviewer response for version 1

**DOI:** 10.21956/openreseurope.16716.r31269

**Published:** 2023-06-26

**Authors:** Tim Tröndle

**Affiliations:** 1 Institute for Environmental Decisions, ETH Zürich, Zürich, Switzerland

The study “ *Global sensitivity analysis to enhance the transparency and rigour of energy system optimisation modelling”* argues for the importance of global sensitivity analyses in energy system optimisation modelling and intends to serve as a guideline on how to perform sensitivity analyses in this context. It presents three energy system models with increasing complexity and applies sensitivity analyses in all of these cases. The study shows how influential parameters can be identified using this method and how inclusion of all model parameters is important.

 I find the study well and transparently written, and I think it can be a valuable contribution to the literature. As the article’s main purpose is pedagogical, I am listing suggested changes related to this purpose below.

1. The parameter uncertainty of model 1 and 2 (and partly model 3) is large and rather unrealistic. I understand you have chosen these ranges to be able to show the effects you are intending to show. However, I tend to think this can be problematic from a pedagogical point of view because input uncertainty quantification is crucial to sensitivity analyses. I understand you may not want to change your chosen ranges, but I would suggest in that case discussing the importance of input uncertainty quantification. 
2. Following my first comment, I would further discuss why input uncertainty quantification is important, namely that all resulting sensitivity analysis findings depend on it. For model 1, you find the most influential parameter to be the one with the largest input uncertainty. Of course there is not a linear relationship between input uncertainty and sensitivity, but there is a relationship, and I tend to think it is crucial for the reader to understand that the results depend on the input uncertainty. I would make this more clear to the reader.
3. Again following, you state that “ *Excluding demand as a sensitivity factor resulted in a Type I error, where we failed to identify an influential factor.”* While this is correct, I tend to think this statement is too absolute as it depends on the input uncertainty and variable of interest or research question. Had you defined smaller input uncertainty (as discussed in my points above) or focussed on, e.g., system LCOE rather than total system cost, the result could be very different. Yes, I acknowledge that you discuss the importance of the choice of output in later sections and the conclusion, but I would suggest making this clear to the reader already here. It may even serve as a transition to the following section.
4. The provision of the Snakemake workflow and input and output data on Zenodo is very valuable in my opinion. However, I tried and was not able to easily reproduce your results. This mainly had to do with the configfile that did not exist (`config/config.yaml` was documented, but I was unable to find it) and me being unsure exactly how I needed to run this. I suggest adding step-by-step instructions that allow readers to reproduce your analyses from the study; especially considering readers that may be unfamiliar with both GSA and Snakemake.
5. Your article has many figures which to me somewhat interrupted the reading flow and of which several have low information content. Even if being explicit in the figures is intended for pedagogical reasons I tend to think that more concise figures are possible, which I would suggest creating.
6. You are using the terms “factor fixing” and “factor prioritisation” on page 5 before introducing them.
7. I do not understand Figures 16 and 22. What is shown on the horizontal axis? Also, Fig 16 has a typo in the label of the vertical axis.
8. The step in Figure 9 confused me. Can you explain what is going on here?
9. On page 17 you quote equation 1 but possibly meant to quote equation 6.

Is the study design appropriate and does the work have academic merit?

Yes

Is the work clearly and accurately presented and does it cite the current literature?

Yes

If applicable, is the statistical analysis and its interpretation appropriate?

Yes

Are all the source data underlying the results available to ensure full reproducibility?

Yes

Are the conclusions drawn adequately supported by the results?

Yes

Are sufficient details of methods and analysis provided to allow replication by others?

Yes

Reviewer Expertise:

energy system analysis

I confirm that I have read this submission and believe that I have an appropriate level of expertise to confirm that it is of an acceptable scientific standard.

# Peer review: Reviewer response for version 1

**DOI:** 10.21956/openreseurope.16716.r30887

**Published:** 2023-05-04

**Authors:** Stefano Moret

**Affiliations:** 1 ETH Zürich, Zürich, Switzerland

The paper provides guidance for considering uncertainty in Energy System Optimization Models (ESOMs) using Global Sensitivity Analysis (GSA) techniques. The first part of the manuscript introduces the concepts of ESOMs and GSA, and illustrates why accounting for uncertainties in energy systems models is important. This is followed by a literature review, demonstrating that only a few studies use SA methods despite the valuable insights they provide. The authors then show how GSA – and more specifically, a SA method informally known as the “Morris method” – can be applied to ESOMs using three different case studies of increasing complexity. This allows them to demonstrate the benefits of applying GSA to ESOMs.

 First, I should mention that I have extensively researched these topics and that my own works are prominently cited in the manuscript. While this does not generate a conflict of interest according to the policy of this journal, I feel compelled to disclose this aspect for transparency reasons.

 My judgment is that this is a well-written paper that addresses an important subject of interest to many researchers in the energy systems domain. While the paper does not present elements of scientific novelty (hence my response “partly” under the academic merit criterion), there is room in the literature for an article like yours that illustrates a known concept with a pedagogical intent if this meets the scope and standards of the journal. As written in the abstract, “the importance of recognizing uncertainty in energy system modeling is regularly commented on but there is little practical guidance on how to best incorporate existing techniques”; I agree with this statement, and I am convinced that this paper can contribute to a broader consideration of uncertainty in energy systems modeling and planning. In particular, using three models at increasing levels of complexity is an effective way to guide the reader step-by-step toward an understanding of the GSA methods and their benefits. The open-source code and data enable the reproduction of the results for pedagogical purposes. 

 Therefore, I recommend that your paper be “approved with reservation” by the journal. I need, however, to stress that the paper does not contain significant elements of scientific novelty; hence, my recommendation holds as long as the journal welcomes “pedagogical” contributions.

 Below are some major and minor comments that the authors should address.

**Major comments:**

1. As the paper prominently relies on the use of the “Morris method,” I was surprised not to find mention of the “scaling issues” affecting this method. Specifically, Sin et al. (2009) [https://doi.org/10.1016/S1570-7946(09)70154-3], a well-known paper in the field, observed that the parameter significance ranking was dependent on the units of the factors used in the model hence leading to erroneous ranking results. To address this issue, they introduced a “scaling factor” based on the ratio of standard deviations to account for different magnitudes of inputs and outputs, which is used, for example, in Moret *et al*, 2017. In Model 3, e.g. in Figure 18, heterogeneous inputs and outputs are considered, yet no scaling seems to be performed. Please address this in the paper and clarify, as this is an important point.
2. Linked to (1), please explain if the mentioned scaling is included in the python library used in this study.
3. Open-source and reproducibility: I commend the authors for the well documented code, which seems to allow reproducibility. Unfortunately, I could not verify this due to time constraints. I recommend that the journal performs a test to confirm that the output results presented in the paper can be reproduced with the provided source code.
4. Your paper is limited to GSA, and even more specifically to the Morris method. Many other methods to consider uncertainty in ESOMs exist (e.g., other SA methods, uncertainty analysis, stochastic programming, robust optimization), with associated advantages and disadvantages. While a detailed overview goes beyond the scope of your work, these options should be briefly presented in the Introduction.

**Minor Comments:**

1. p. 3: you state that the “adoption of GSA is underrepresented”. Can you backup this statement with numbers? For example, at p. 6, you mention a study by Yue *et al* (2018), reviewing 2100 uncertainty studies. This seems high, and checking the reference this number appears to be the total number of reviewed studies before applying any filter. Readers may be interested in the following questions: what percentage of ESOMs studies consider uncertainty? How many of these use SA / GSA? Please address these. 
2. p. 3: “MIP are solved approximately”: MILP / MIP can be solved to global optimality; please review this statement.
3. p. 5: “This prior step is often called uncertainty analysis”: do you mean “uncertainty quantification” here? “Uncertainty Analysis” is normally used to describe methods focusing on the outputs of a model.
4. p. 6: “UQLab for Matlab”: the group recently released a python version, please update this statement. 
5. p. 7: “using Morris”: in that paper, both Morris and variance-based methods are used.
6. p. 8: “arbitrary ±X% ranges”: estimating a ± X% range is not necessarily arbitrary. Often, in ESOMs, the quantification of uncertainty is challenging and it is difficult to come up with anything beyond upper / lower bounds for uncertain parameters. Please clarify. 
7. Figures 1-3: I suggest referring to these models as “Model 1”, “Model 2” and “Model 3” in the captions, so that it is clear for the reader.
8. Figure 3: what does “Mine” refer to in this figure?
9. p. 11: “time-independent or time-independent”: a similar classification was introduced (Type I, II, III) in Moret *et al*, 2017, which you already cite in other sections of the paper. Please comment on this here.
10. In Figure 9 we see a step-change in capital investments which we do not see in Figure 7. Please explain.
11. Table 5: Why are fixed costs expressed in M$/GW and not in M$?
12. Table 5: The uncertainty range for demand, [19, 122] PJ, seems extremely large / unrealistic. This might influence the importance of demand in your results. Why did you choose such a large range?

Is the study design appropriate and does the work have academic merit?

Partly

Is the work clearly and accurately presented and does it cite the current literature?

Yes

If applicable, is the statistical analysis and its interpretation appropriate?

Yes

Are all the source data underlying the results available to ensure full reproducibility?

Yes

Are the conclusions drawn adequately supported by the results?

Yes

Are sufficient details of methods and analysis provided to allow replication by others?

Yes

Reviewer Expertise:

Energy Systems Modelling, Optimization, Uncertainty

I confirm that I have read this submission and believe that I have an appropriate level of expertise to confirm that it is of an acceptable scientific standard, however I have significant reservations, as outlined above.
